# A partial Drp1 knockout improves autophagy flux independent of mitochondrial function

**DOI:** 10.1186/s13024-024-00708-w

**Published:** 2024-03-19

**Authors:** Rebecca Z. Fan, Carolina Sportelli, Yanhao Lai, Said S. Salehe, Jennifer R. Pinnell, Harry J. Brown, Jason R. Richardson, Shouqing Luo, Kim Tieu

**Affiliations:** 1 Department of Environmental Health Sciences, Florida International University, Miami, USA; 2 Biomolecular Sciences Institute, Florida International University, Miami, USA; 3 Peninsula Schools of Medicine and Dentistry, Plymouth University, Plymouth, UK

**Keywords:** Parkinson’s disease, Manganese, Mitochondrial dynamics, Mitochondrial dysfunction, Dynamin related protein 1, Autophagy, Protein aggregation, α-synuclein

## Abstract

**Background:**

Dynamin-related protein 1 (Drp1) plays a critical role in mitochondrial dynamics. Partial inhibition of this protein is protective in experimental models of neurological disorders such as Parkinson’s disease and Alzheimer’s disease. The protective mechanism has been attributed primarily to improved mitochondrial function. However, the observations that Drp1 inhibition reduces protein aggregation in such neurological disorders suggest the involvement of autophagy. To investigate this potential novel protective mechanism of Drp1 inhibition, a model with impaired autophagy without mitochondrial involvement is needed.

**Methods:**

We characterized the effects of manganese (Mn), which causes parkinsonian-like symptoms in humans, on autophagy and mitochondria by performing dose-response studies in two cell culture models (stable autophagy HeLa reporter cells and N27 rat immortalized dopamine neuronal cells). Mitochondrial function was assessed using the Seahorse Flux Analyzer. Autophagy flux was monitored by quantifying the number of autophagosomes and autolysosomes, as well as the levels of other autophagy proteins. To strengthen the in vitro data, multiple mouse models (autophagy reporter mice and mutant Drp1^+/−^ mice and their wild-type littermates) were orally treated with a low chronic Mn regimen that was previously reported to increase α-synuclein aggregation and transmission *via* exosomes. RNAseq, laser captured microdissection, immunofluorescence, immunoblotting, stereological cell counting, and behavioural studies were used.

**Results in vitro:**

data demonstrate that at low non-toxic concentrations, Mn impaired autophagy flux but not mitochondrial function and morphology. In the mouse midbrain, RNAseq data further confirmed autophagy pathways were dysregulated but not mitochondrial related genes. Additionally, Mn selectively impaired autophagy in the nigral dopamine neurons but not the nearby nigral GABA neurons. In cells with a partial Drp1-knockdown and Drp1^+/−^ mice, Mn induced autophagic impairment was significantly prevented. Consistent with these observations, Mn increased the levels of proteinase-K resistant α-synuclein and Drp1-knockdown protected against this pathology.

**Conclusions:**

This study demonstrates that improved autophagy flux is a separate mechanism conferred by Drp1 inhibition independent of its role in mitochondrial fission. Given that impaired autophagy and mitochondrial dysfunction are two prominent features of neurodegenerative diseases, the combined protective mechanisms targeting these two pathways conferred by Drp1 inhibition make this protein an attractive therapeutic target.

**Graphical Abstract:**

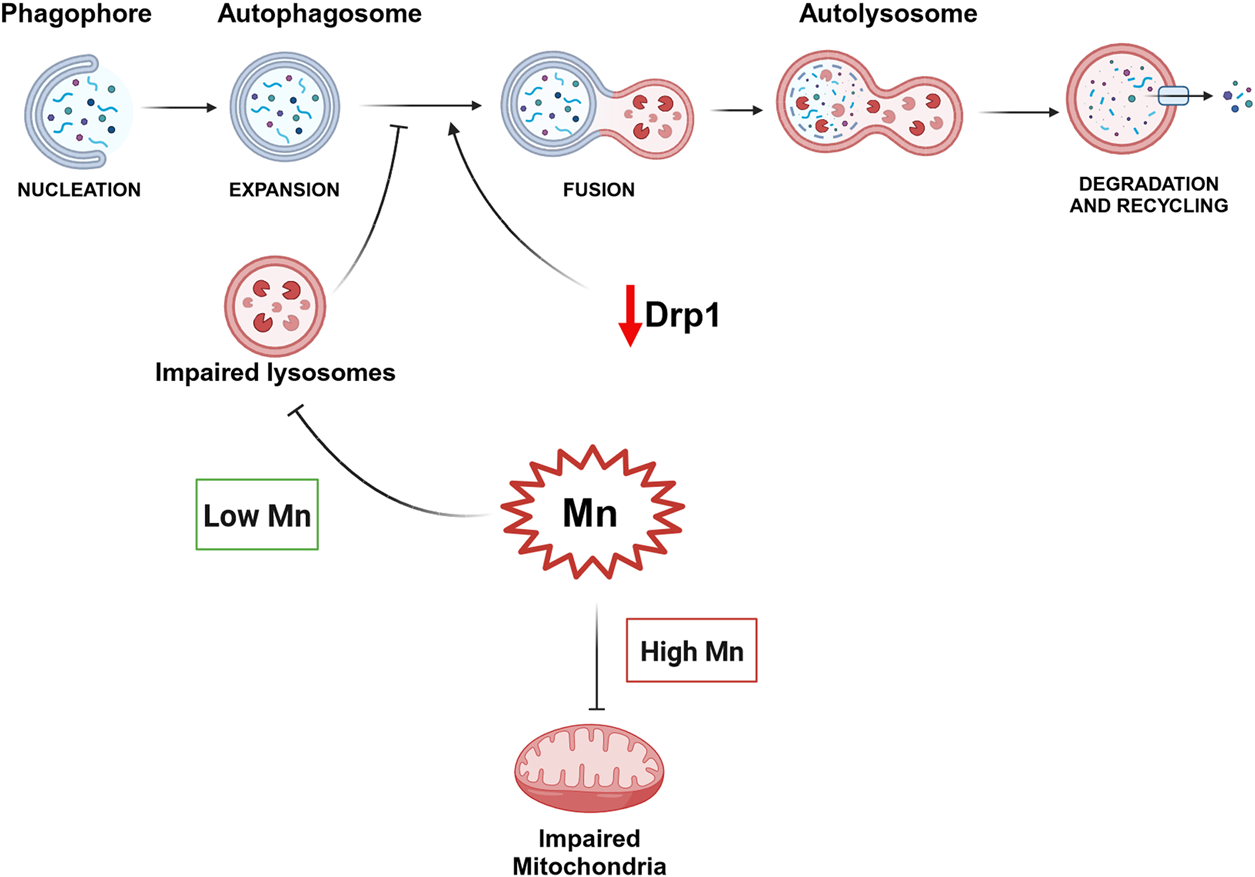

**Supplementary Information:**

The online version contains supplementary material available at 10.1186/s13024-024-00708-w.

## Background

Dynamin-related protein 1 (Drp1) is a member of the dynamin GTPase superfamily. Mutations in Drp1 cause severe neurologic phenotypes in humans [1]. This protein has four functional domains [2]. The N-terminal GTPase domain is essential for GTP hydrolysis. The middle domain is important for Drp1 self-assembly into dimers, tetramers, and higher-order oligomeric structures. The C-terminal GTPase-effector (GED) domain is important for mediating both intra- and intermolecular interactions of Drp1 proteins. Lasty, the small insert variable or B domain, whose function has not been fully resolved; but it has been reported to possess a lipid-binding region [3], which is consistent with the observation that Drp1 binds to the outer mitochondrial membrane cardiolipin under stress conditions to induce mitochondrial fragmentation [4]. Overall, Drp1 is best known for its role in mitochondrial fission [5, 6]. Upon activation in mammalian cells, Drp1 translocates from the cytosol to the outer mitochondrial membrane, where it binds to mitochondrial fission factor (Mff), Fis1, and mitochondrial division proteins 49 and 51 (MiD49 / MiD51). Drp1 self-assembles into spiral structures which constrict and divide the mitochondrion into two daughter organelles [5, 6].

Although the role of Drp1 in mitochondrial division is critical to normal cellular function, excessive mitochondrial fission is detrimental to cells. Accumulating evidence indicates that partial Drp1 inhibition is protective in experimental models of neurodegenerative diseases such as Parkinson’s disease (PD), Alzheimer’s disease (AD), Huntington’s disease (HD), and Amyotrophic Lateral Sclerosis (ALS) [5, 6]. The protective mechanism of blocking Drp1 function in these studies has been largely attributed to restoring the balance in mitochondrial fission and fusion. However, despite its widely documented role in mitochondrial fission, Drp1 predominantly resides in the cytosol with only approximately 3% cofractionating with mitochondria [7]. Live-cell imaging of fluorescently labelled Drp1 reveals that much of Drp1 appears diffuse in the cytoplasm and only approximately 2.5% of the total Drp1 puncta engage in mitochondrial fission [8]. Such observations, combined with reports that Drp1 also colocalizes with other organelles such as lysosomes [9], endoplasmic reticulum [10] and peroxisomes [11], suggest that Drp1 most likely is a multifunctional protein that exerts its effects beyond mitochondrial fission. Indeed, Drp1 inhibition has been reported by independent laboratories to reduce protein aggregation in experimental models of PD [12, 13], AD [14, 15] and HD [16], indicating the potential involvement of protein removal pathways such as autophagy. However, because these models impair mitochondrial function, which may result in impaired autophagy, it is not feasible to untangle with certainty whether Drp1 inhibition reduces protein aggregation via mitochondria, autophagy or a combination of both. A model with impaired autophagy independent of mitochondrial involvement is needed to evaluate this potential novel protective mechanism of Drp1 inhibition.

Manganese (Mn) is an essential trace metal required for normal development and function because it is essential for the activity of a variety of enzymes, including glutamine synthetase and hydrolases, and it is a cofactor for a variety of metalloproteins, including the mitochondrial Mn-superoxide dismutase (MnSOD) [17–19]. However, excessive exposure to Mn can cause parkinsonian-like symptoms known as manganism. Although symptoms resemble those of PD, manganism and PD are two distinctive disorders [20–22]. Nevertheless, both human and experimental data support the notion that chronic low-level Mn exposure represents a risk factor for developing PD, as well as accelerating the disease onset and progression of PD by interacting with an individual’s susceptible genes [23–26]. Indeed, higher blood levels of Mn has been reported in some PD patients [27]. Experimentally, chronic exposure to Mn in rat results in increased Mn levels in dopamine (DA) neurons of the substantia nigra *pars compacta* (SNpc) [28]. In combination, evidence suggests that Mn may contribute to PD pathogenesis.

The link between Mn toxicity and mitochondrial dysfunction has been made since the early observations that at high concentrations, Mn enters mitochondria through the Ca^2+^ uniporter channel [29] and accumulates in the mitochondrial matrix, resulting in impaired oxidative phosphorylation [30]. However, the negative impact of Mn on mitochondria below cytotoxic concentrations has been questioned in a recent study [31]. Furthermore, under conditions where Mn induces mitochondrial dysfunction, autophagy is also affected [32]. Given the bi-directional relationship between mitochondria and autophagy [33], it is not clear whether mitochondrial dysfunction precedes autophagy blockade or vice versa for Mn neurotoxicity. Combined with the observation that Mn is accumulated to a greater extent in lysosomes than in mitochondria [34], it is critical to investigate whether autophagy or mitochondria is the initial vulnerable target of Mn.

In this study we provide data to address two major gaps of knowledge in the field: First, by titrating the doses of Mn and by using multiple cell and animal models, it is evident that autophagy flux, not mitochondria, is the target of low level Mn exposure. Second, using Mn as in vitro and in vivo models to impair autophagy without affecting mitochondria, a partial Drp1 knockout improved autophagy flux, indicating this protective mechanism is mediated independent of mitochondria.

## Methods

### Cell cultures

HeLa cells with stable expression of mRFP-GFP-LC3 were used as autophagy reporter cells [13]. These cells were grown in Dulbecco’s Modified Eagle’s Medium (DMEM, Gibco) supplemented with 10% Fetal Bovine Serum (FBS, Gibco), 100U/ml penicillin/ 100 µg/ml streptomycin and 100 µg/ml G418 (Gibco) at 37 °C in 5% CO_2_.

Immortalized rat dopaminergic N27 neuronal cells with stable and inducible expression of human WT α-synuclein were generated using the ecdysone-inducible system as previously described [13]. They were grown in RPMI1640 medium, supplemented with 10% FBS, 500 µg/ml G418 and 200 µg/ml Hygromycin B (Gibco) at 37 °C in 5% CO_2_. To induce α-synuclein expression, cells were treated with 20 µM ponasterone A (an ecdysone-homolog).

### Cytotoxicity using calcein AM

HeLa cells were plated at a density of 6 × 10^3^ cells/well (for 24 h treatments) or 4 × 10^3^ cells/well (for 48 h treatments) in 96-well plates, respectively. N27 cells were plated at 1 × 10^4^ cells/well (for 24 h treatments) and 8 × 10^3^ cells/well (for 48 h treatments). Cells were treated with varying concentrations (62.5µM to 2mM) of MnCl_2_ for 24 or 48 h. Cytotoxicity was assessed using Calcein AM assay (Thermo Fisher Scientific) in accordance with the manufacture’s protocol. Fluorescent signal from the Calcein AM dye was measured using a plate reader (Synergy H1, Biotek).

### Cell transfection and siRNA-mediated Drp1 knockdown

Drp1 knockdown in HeLa and N27 cells were performed as previously described [13]. Briefly, pre-designed siRNA against human *DNM1L* (gene encoding Drp1) and rat *Dnm1l* were purchased from Dharmacon Research, Inc (now available through Horizon Discovery). SMARTpool: siGENOME Human *DNM1L* siRNA (Cat# 10,059) was used for HeLa cells, while SMARTpool: siGENOME Rat *Dnm1l* siRNA (Cat# 114,114) was used for N27 cells. Each of this product is a mixture of four siRNA targeting a single gene to enhance efficiency and specificity of Drp1-knockdown. Similarly, the scramble control, siGENOME Non-Targeting siRNA Control Pools (Cat# D-001206, Dharmacon Inc.), was designed to have a minimum of four mismatches to all human, mouse and rat genes, and confirmed by the manufacturer to have minimal targeting by genome-wide microarray analysis. To monitor LC3 levels in N27 cells, mCherry-hLC3B-pcDNA3.1 plasmid was used. All the transfection was performed using Lipofectamine™ 3000 (Thermo Fisher Scientific) according to the manufacturer’s instructions.

### Immunostaining

*Cultured cells*. Cells were grown on borosilicate cover slips pre-coated with poly-D-lysine in 24-well plates. Prior to immunostaining, cells were fixed with 4% formaldehyde (Thermo Fisher Scientific), in warm cell culture media at 37 °C for 20 min. After washing with 0.1 M phosphate buffer saline (PBS), cells were incubated with 4% normal goat serum (NGS) (Vector Laboratories), 0.1% TritonX-100 in 0.1 M PBS for 1 h at room temperature before being processed for immunostaining. The primary antibodies used were rabbit anti-TOM20 (1:500, cat. no. FL-145, Santa Cruz), mouse anti-Drp1 (1:500, cat. no. 611,113, BD Biosciences), rabbit anti-p62/SQSTM1(1:500, cat. no. PM045, MBL), rabbit anti- α Synuclein antibody (1:500, cat. no. AB5038, Millipore). Corresponding Alexa Fluor® (350, 488, 586, and 633) conjugate secondary antibodies (Invitrogen) were used at 1:500-1:1000 dilution. Samples were incubated in primary antibodies overnight at 4 °C, and in secondaries at room temperature for 1 h, with three washes in 0.1 M PBS in between all incubation steps carried out on an orbital shaker. The cover slips were then mounted on to slides using Prolong™ gold anti-fade mount with or without DAPI (Thermo Fisher Scientific).

*Brain sections*. Mice were perfused with 0.9% saline followed by 4% PFA (prepared in 0.1 M phosphate buffer), the brains were removed and further fixed with 4% PFA overnight at 4ºC. Sequential overnight incubations in 15% and 30% sucrose in 0.1 M phosphate buffer were conducted before the brains were then snap frozen in 2-methylbutane at -55 °C. Frozen brains were mounted in optimal cutting temperature (OCT) medium, and cryo-sectioned at 30 μm using cryostat (CryoStar NX70, Thermo Fisher Scientific). Coronal sections were collected free-floating in 0.1 M TBS. Sections from mid brain were washed in 0.1 M TBS before non-specific binding sites were blocked in 0.1 M TBS, 4% NGS, 0.1% TritonX-100 for 1 h at room temperature. Primary antibodies diluted in 0.1 M TBS, 2% NGS, 0.1% TritonX-100 were then added and incubated overnight at 4 °C. Following washing, sections were incubated with Alexa Fluor® secondary antibodies (1:1000, Invitrogen), diluted in 0.1 M TBS, 2% NGS, for 1 h at room temperature protected from light. Primary antibodies used were chicken anti-tyrosine hydroxylase (1:2000, cat. no. 76,442, Abcam), mouse anti-GAD67 (1:500, cat. no. MAB5406, Millipore), rabbit anti-TOMM20 (1:1000, cat. no. ab186735, Abcam). Following immunostaining with antibodies, sections were stained with 5ug/ml DAPI (cat. no. 62,247, Thermo Scientific™) for 10 min and washed three times before mounted onto microscope slides, cover glassed with Prolong™ gold anti-fade mount (Thermo Fisher Scientific).

All fluorescent images were captured using Olympus Fluoview (FV1200) confocal microscope with 60x or 100x objectives, lasers 405 nm, 473 nm, 559 nm, 635 nm were set at 5–6% for all channels.

### Quantification of autophagic vesicles

The analysis of autophagy flux in stable HeLa cells expressing mRFP-EGFP-LC3 was performed as previously described [13]. Red and green fluorescent vesicles in cells were imaged using confocal microscopy with 60x time objective. Autophagosomes and autolysosomes from at least 50 cells per group were quantified using Fiji. For N27 cells, autophagy blockage was assessed by quantifying confocal captured LC3-mcherry puncta and immunostained p62 puncta using Imaris 3-dimensional (3D) analysis software (Oxford instruments) as previously described [35, 36]. 3D rendering of cells was made using the “Surfaces” function with the appropriate threshold to allow total cell volume calculations. Number of puncta was normalized to the cell volume.

### Lysosomal pH quantification

Change in lysosome pH was measured using LysoSensor™ Yellow/Blue DND-160 (Molecular Probes) with a plate reading method adapted from a previous publication [37]. N27 cells were treated with varying concentrations of Mn for 24 h. Bafilomycin A1 (400nM, 4 h) was used as positive control to increase lysosomal pH. Cells were then trypsinized, counted, and per 1 × 10^6^ cells were resuspended in 100 µl Lysosensor working solution (1 µM diluted in DMEM media), incubated at 37 °C for 5 min, washed 3X with HBSS, and resuspended in 1 ml of HBSS. Cell suspension (150 µl) was added into each well of a black 96-well plate, and fluorescent signal was measured using SepctraMax M5 (Molecular devices). The dye was excited at 355 nm, and emission wavelengths at 452 nm and 521 were recorded. The ratio of emissions at 521/452 nm was calculated to reflect the changes of lysosomal pH from acidic to neutral. A decreased ratio indicates an increase in lysosomal pH.

### Mitochondrial network analysis (MiNA)

To assess the effect of Mn exposure on mitochondrial network/morphology, Mitochondrial Network Analysis (MiNA, a macro toolset of Fiji utilizing existing ImageJ plug-ins) was performed as described [38]. Briefly, mitochondria were visualized by immunofluorescence using TOM20 (Abcam, ab186735) and imaged with the Olympus Fluoview 1200 confocal. Prior to MiNA analysis, 8-bit gray scale images were binarized and skeletonized following the steps of unsharp mask, enhance local contrast (CLAHE), and median filtering using Fiji. After generating morphological skeletons (2D), the “tagged skeletons” were analyzed using MiNA, which divides objects (mitochondria) into two distinct types: individuals (puncta and rods but no branches) and networks (with connected branches). “Mean branches per Network” represents the average number of branches per network. “Mean rod/branch length” calculates the average length of all rods and branches.

### Mitochondrial membrane potential

Mitochondrial membrane potential assessment was performed as described in our previous publication [39]. Briefly, N27 cells were treated with 125 µM Mn for 24 h, washed and detached, pellets were resuspended and incubated in HBSS containing 50 nM tetramethylrhodamine methyl ester (TMRM). Carbonyl cyanide 4-(trifluoromethoxy) phenylhydrazone (FCCP, 20 µM) was used as positive control to collapse ΔΨm. BD Accuri C6 flow cytometer was used for fluorescence analysis.

### Proteinase K digestion

To determine the formation of insoluble protein aggregation in N27 cells, after 4% PFA fixation, cells were incubated with 5U/ml PK solution for 10 min at 37 ºC, then 10 min at room temperature (˜23 ºC) with gentle shaking. After 3 × 5 min washed with PBS, cells were immunostained with α-synuclein (Millipore, AB5038). Following confocal imaging, the number of insoluble aggregates was quantified using Imaris software as described above. The same technique was applied to reconstruct nucleus volume based on DAPI signal. Individual PK-resistant aggregates was determined by thresholding spheres that colocalize with immunofluorescent staining. The cytosolic aggregate number was normalized to cytoplasmic volumes (total cell volume – nuclear volume). The average number of puncta was determined by counting approximately 25 cells per sample with three independent replicates.

### Mitochondrial respiration assay

Mitochondrial respiration was assessed using Agilent XF^e^ 96 Extracellular Flux Analyzer (Agilent Inc) as previously described [13]. Briefly, cells were plated onto XF Cell Culture Microplate and treated with different concentrations of MnCl_2_ for 24 h. The culture media was then replaced with XF assay media (XF base media supplied with 1mM pyruvate, 2mM glutamax, 2mM glucose and 2mM HEPES, pH 7.2), the plate was then acclimatized in a non-CO_2_ incubator for 30 min and placed in the Analyzer. A standard XF cell mito stress test with sequential injections of Oligomycin, FCCP and Rotenone/Antimycin was conducted.

### Gel-free immunoblotting

Gel-free immunoblotting was performed with Jess™ system (ProteinSimple, Bio-Techne) using 12–230 kDa Fluorescence Separation Module (cat. no. SM-FL004 ProteinSimple, Bio-Techne), as per manufacturer’s instructions. Cultured cells or brain samples were lysed using 1X RIPA buffer with proteinase inhibitor cocktail (Thermo Fisher Scientific) prior to centrifugation at 13,000*g* (4 °C, 15 min) to collect supernatant. After BCA protein quantification (Thermo Fisher Scientific), lysates were diluted in 0.1X sample buffer (included in the separation module) and mixed with 5x Fluorescent Master Mix (PS-ST01EZ, ProteinSimple, Bio-Techne) to a final concentration of 0.5 µg/µl. Samples were then boiled at 95 °C for 5 min or 50 °C for 30 min (for p62), and 1.5 µg of protein were loaded for each sample. The primary antibodies used were rabbit anti-Atg5 (1:100, cat. no. NB110-53818, Novus Biologicals, Bio-Techne), rabbit anti-Drp1 (1:100, cat. no. NB110-55237, Novus Biologicals, Bio-Techne) and rabbit anti-p62 (1:50, cat. no. NBP1-48320, Novus Biologicals, Bio-Techne). Anti-mouse and anti-rabbit secondary antibodies were prepared using corresponding detection modules from ProteinSimple (Bio-Techne) following manufacturer’s instructions. For normalization, Total Protein Detection Module (DM-TP01) were used in combination with RePlex™ Module (RP-001, ProteinSimple, Bio-Techne). Data were recorded and analyzed using Compass v6.0.

### Animal models and Mn treatment

All mice in this study were bred, maintained, and characterized at animal care facility of Florida International University (FIU). Animal care and procedures were approved and conducted in accordance with Institutional Animal Care and Use Committee at FIU. C57BL/6-Tg(CAG-RFP/GFP/Map1lc3b)1Hill/J mice (#027139) were purchased from the Jackson Laboratory. These transgenic autophagy reporter mice express ubiquitously a tandem RFP-EGFP-LC3 fusion protein under the CAG promoter [40]. Hemizygotes were crossed with C57BL/6J (Jackson Laboratory, #000664) mice to establish and maintain the colony.

*Dnm1l*-deficient mice (Dnm1l^tm1b/tm1c (KOMP) wtsi/Ics^, Institut Clinique de la Souris, France). We contracted the European Conditional Mouse Mutagenesis Consortium (EUCOMM) to generate Drp1-knockout (KO) mice. These animals were generated using “knockout first” technology [41] in C57BL/6 N embryonic stem cells [42]. As described by the mouse producer, this strategy relies on the identification of a ‘critical’ exon common to all transcript variants that, when deleted, creates a frame-shift mutation. The KO-first allele is flexible and can produce reporter knockouts, conditional knockouts, and null alleles following exposure to site-specific recombinases Cre and Flp (Please refer to the illustration in Fig. 7A). The ‘knockout-first’ (tm1a) mice, which contain an IRES:*lacZ* trapping cassette and a floxed promoter-driven *neo* cassette inserted into the intron of the *Dnm1l* gene, were crossed with non-specific cre mouse strain to delete the promoter driven cassette and floxed exon of the *Dnm1l* allele. This generates a ‘global’ *lacZ* tagged allele expressing mouse strain that lacks Drp1 in all cell types (tm1b mouse). As the homozygous disruption of Drp1 is known to be embryonically lethal, we maintain the tm1b strain as a global heterozygous Drp1-KO (Drp1^+/−^) by crossing with C57BL/6J mice. These mice are viable, fertile and do not display any identifiable defects compared to control mice as demonstrated in. Mice were backcrossed more than 10 generations to completely switch the background to C57BL/6J before Mn treatment was carried out.

*Mn treatment*. 3-month-old mice were orally gavaged with either water control or 15 mg/kg/day of MnCl_2_;·4H_2_O (cat. no 221,279, Sigma-Aldrich), equivalent to ˜ 4.2 mg absolute Mn/kg/day, once daily for 30 consecutive days as previously described [43]. Mice were sacrificed one day after the last treatment. Brains were micro-dissected by regions and snap-frozen for further analysis.

### RNA-Seq and transcriptomic analysis

Total RNA was extracted from mouse ventral midbrain using TRIzol (Thermo Fisher Scientific)) as per manufacturer’s instructions. RNA quality evaluation, cDNA library construction and Illumina sequencing were performed by Novogene Corporation Inc. Differential expression analysis was conducted using the DESeq2 R package (1.20.0) and applied Benjamini Hochberg multiple test correction. Genes with adjusted *p*-value < 0.05 and absolute log_2_(FoldChange) > 0 were considered as differentially expressed. The gene enrichment analysis of the differentially expressed genes (DEGs) that are specifically associated with autophagy pathways were performed using the “Autophagy Transcription Gene Toolbox” [44]. Kyoto Encyclopedia of Genes and Genomes (KEGG) pathway analyses were further performed to validate the enrichment of autophagy pathways [45]. The DEGs that are involved in mitochondrial dynamics and mitochondrial function were also compared between the Mn-treated mice and the control mice in reference to “Mouse Genome Informatics” and “Mouse MitoCarta3.0”, respectively [46, 47].

### Quantitative RT-PCR

For RT-PCR, 1 µg of total RNA was converted to complementary DNA (cDNA) using iScript Reverse Transcription Supermix (Bio-Rad). qPCR was conducted on a QuantStudio 6 detection system (Thermo Fisher Scientific) using TaqMan assays and TaqMan Fast Advanced Master Mix (Thermo Fisher Scientific). The Taqman assays employed were *Dnm1l* (mouse, assay ID: Mm01342903_m1, Thermo Fisher Scientific) and *Gapdh* (mouse, assay ID: Mm99999915_g1, Thermo Fisher Scientific). *Gapdh* served as the internal control. The reaction conditions were as follows: 50 °C for 2 min, 95 °C for 2 min and 40 cycles of 95 °C for 1 s and 60 °C for 20 s. Relative quantification was performed using the 2^−ΔΔCT^ method.

### Mouse body weight and length measurements

The monitoring of mice throughout their development allowed for comparison of growth parameters through various stages of development. At each measurement, bodyweight was recorded along with the length of each animal. To enable standardized estimation of mouse length; in brief, the mouse was placed on laminated graph paper (5 mm x 5 mm grid size) and when in a relaxed position a photo was taken directly from above. The images were collated and labelled with mouse ID prior to quantification of the mouse length. Using Fiji, the scale was set for each image using the 5 mm grid. The use of this grid allowed accurate scale determination regardless of variance in the height from which the image was taken. A midline was then drawn along the center of the mouse and the length measured along the midline. For weight measurement, mice were habituated into the test room for 10–20 min and weighed on the electronic scale with a perforated cover.

### Behavioral studies

3-4-month-old Drp1^+/−^ mice and littermate controls were used for all behavioral assessments. For locomotor activity recordings, mice were habituated to the testing room for half an hour before testing. Animals were placed into the activity chamber (Activity Cages Monitor SOF-812, Med Associates, Fairfax, VT) and monitored for 60 min using activity monitor software (Activity Monitor version 7.0.5.10 SOF-812, Med Associates). All locomotor activity recordings were performed during the dark cycle (after 19:00 pm) when mice were active. Novel Object Recognition test was used to assess learning and memory in mice. Naïve mice were habituated to the rectangle testing chamber for one hour. Twenty-four hours later, mice were placed back into the same chamber with two identical objects and allowed to explore the objects for 10 min before returning to the home cage. One hour later, they were returned to the same chamber with one object replaced by a novel object and allowed to explore the objects for 5 min while being recorded using Nodulus software. The recognition index was calculated as a fraction of the time taken to explore the novel object to the total exploration time. Typically, mice with intact learning and memory capacity spend more time exploring novel objects than familiar ones.

### Quantification of nigral DA neurons

Stereological cell counting was performed as described [48]. Briefly, serial coronal sections (30 µM) were collected free-floating and every 4th midbrain section spanning caudal to rostral was incubated with anti-tyrosine hydroxylase (1:4000, cat. no. 657,012, Millipore), followed by biotinylated goat anti-rabbit IgG (1:200 dilution; cat. no. BA-1000, Vector Laboratories), and avidin-biotin complex (Vectastain® ABC HRP Kit, Vector Laboratories). Immunoreactivity was visualized using diaminobenzidine (DAB). Total numbers of TH-positive neurons in substantia nigra par compacta were counted stereologically using the optical fractionator (Visiopharm stereological software, Denmark). The settings for the optical dissector probe (dissector height and counting frame size) were set up at 100x objective (Olympus, UPlanApo, NA 1.35, oil iris). A sampling percentage of 30% of the ROI was counted, using a counting frame area of 6400 μm (80 μm x 80 μm). A guard zone of 1 μm was utilized at the upper and lower limits of the dissector. The CE values were < 0.1 for all animals.

### Immunofluorescence-laser microdissection

Serial coronal nigral Sect. (20-µm) were cut on a standard cryostat (CryoStar NX70, Thermo Fisher Scientific) with a clean blade from snap-frozen mouse brain tissue (one hemisphere). Six to eight sections from each brain were mounted on PEN membrane slides (4 μm, cat. no. 11,600,288, Leica), and the unfixed sections were immediately stored at -80 °C until immunofluorescence-LMD was performed. The frozen sections were thawed at room temperature for 1 min without drying and immersed immediately in cold acetone for 5 min. After fixation, the sections were rinsed briefly in 1xPBS, pH 7.4, and subjected to immunostaining. The sections were initially blocked with 5% NGS for 30 min, followed by incubation with primary antibodies for 1 h and secondary antibodies for 30 min; all steps were performed at room temperature. Sections were briefly rinsed with 1x PBS between each step. After counterstaining with DAPI for 5  min at room temperature, the sections were dehydrated in graded alcohols (30 s each) and air-dried. The primary antibodies used were rabbit anti-tyrosine hydroxylase (1:250, cat. no. 657,012, Millipore) and mouse anti-GAD67 (1:50, cat. no. MAB5406, Millipore). The secondary antibodies used were Alexa Fluor 568 goat anti-rabbit (cat. no. A11011, Thermo Fisher Scientific) and Alexa Fluor 488 goat anti-mouse (cat. no. A11029, Thermo Fisher Scientific). Both secondary antibodies were used at 1:100 dilution. Subsequently, the immunostained DA neurons in the substantia nigra *pars compacta* and GABA neurons in the substantia nigra *pars reticulata* were collected by laser microdissection (LMD6, Leica). The microdissected tissues (2-4mm^2^) were further lysed in 1xSDS lysis buffer (1% (w/v) SDS, 10mM EDTA, pH 8.0, and 50mM Tris-HCl, pH 8.0) prior to gel-free immunoblotting for p62.

### Statistics

All values are expressed as mean ± SEM. Differences between means were analysed using one- or two-way ANOVA with different mouse genotypes, treatment or time as the independent factors. When ANOVA shows significant differences, Tukey’s post-hoc test was used for pair-wise comparisons between means. All data sets were subjected to a normality test and an equality of variance test. When these criteria were not met, nonparametric analysis was applied (Kruskal-Wallis ANOVA test with Dunn’s post-hoc test). In all analyses, the null hypothesis (H_0_) or the alternative hypothesis (H_1_) was accepted with an a-error (false-positive) ≤ 5% and a b-error (false-negative) ≤ 20%, respectively.

## Results

### Mn impairs autophagy at low concentrations

**Fig. 1:**
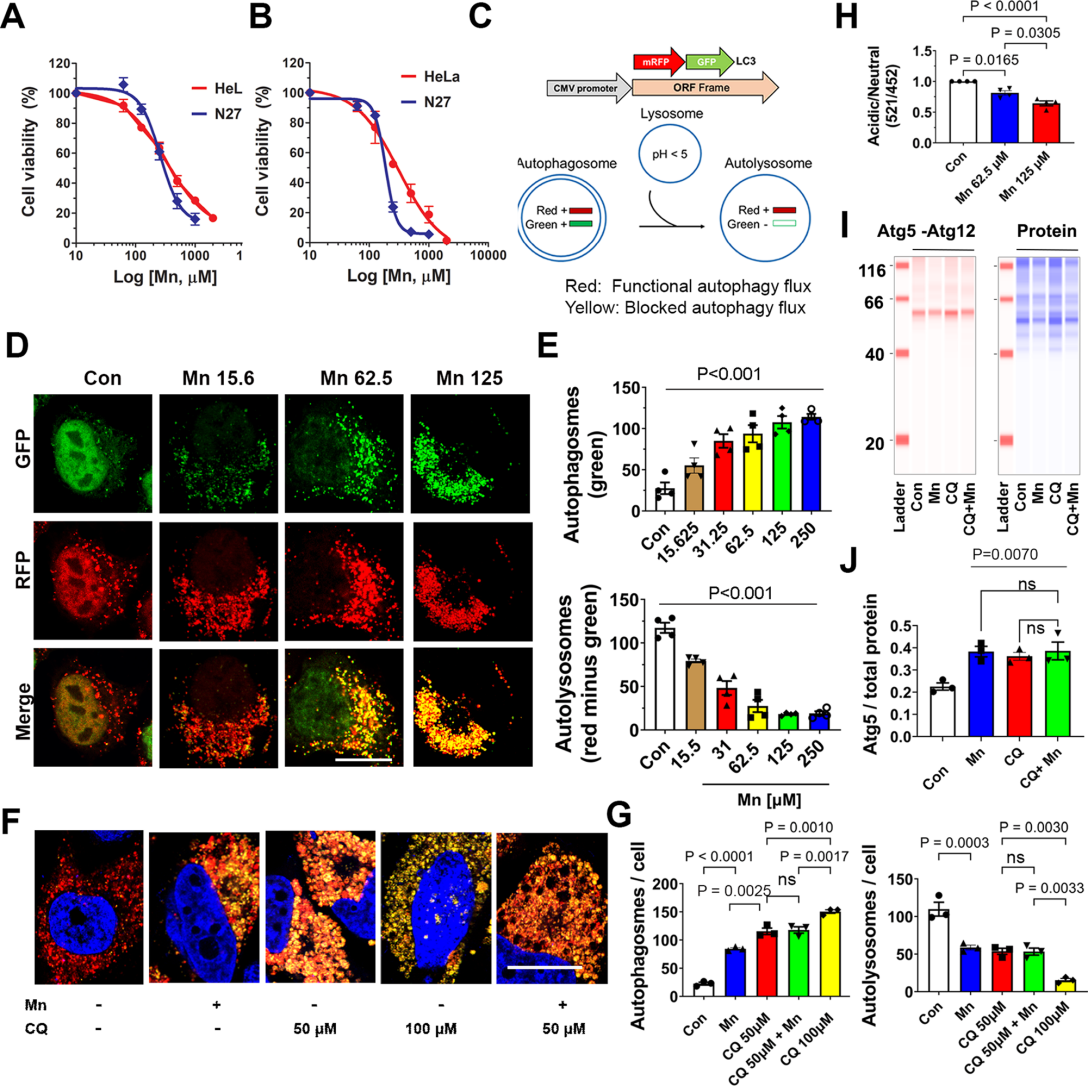
Dose-responses of Mn on cell viability and autophagy flux. (**A**) Stable HeLa cells overexpressing mRFP-GFP-LC3, and immortalized rat dopaminergic neuronal cells N27 were exposed to MnCl_2_ (62.5 µM to 2mM) for 24 h, or (**B**) 48 h. Cell viability was evaluated using Calcein AM dye. Data represent mean ± SEM, *n* = 3 independent experiments with 4 replicates per experiment. (**C**) Schematic diagram illustrating the autophagy flux pathway and the construct used to create the mRFP-GFP-LC3 stable HeLa reporter cells. With this cell model, autophagosomes appear yellow due to the colocalization of RFP and GFP signals. Red signal indicates the flux is functional because the green signal is quenched by the acidic environment of the lysosomes, which fuse with autophagosomes. These stable cells were treated with vehicle control or Mn (15.6–250 µM) for 24 h. (**D**) Representative images of cells treated with different doses of Mn were captured using confocal microscopy. (**E**) Green and red vesicles per cell were quantified using Fiji. Green vesicles represent autophagosomes. The number of autolysosomes was calculated by subtracting the number of green from the red puncta per cell. Scale bar = 20 μm. (**F**) Stable HeLa cells were treated with MnCl_2_ (125 µM), chloroquine (CQ) or both. (**G**) The number of autophagosomes and autolysosomes were quantified. The combination of Mn with CQ did not significantly alter the numbers of autophagosome (*P* = 0.9941) and autolysosome(*P* > 0.9999), as compared to CQ (50 µM) only, a dose that did not completely inhibit autophagy. Data represent mean ± SEM. (**H**) N27 cells were treated with 62.5 or 125 µM of MnCl_2_ for 24 h, then incubated with LysoSensor Yellow/Blue. The ratio of acidic (yellow) vs. neutral (blue) pH of lysosomes were quantified using a plate reader. (**I**) N27 cells were treated with Mn 125 µM, CQ50 µM or both for 24 h, followed by immunoblotting for Atg5, a marker for autophagosomes, using Jess (ProteinSimple, Inc.) (**J**) Total protein per capillary was used as loading control to normalize the levels of Atg5. Mn did not further increase the accumulation of autophagosomes when combined with CQ versus CQ alone (*P* = 0.9998). All data represent mean ± SEM (*n* = 3–4 independent experiments with approximately 30 cells per experiment for **E** and **G**) and groups were compared by using Kruskal-Wallis ANOVA test with Dunn’s post-hoc test; ns: non-significant

To investigate whether mitochondria or autophagy is the initial vulnerable target to Mn, a systematic approach is needed to characterize the impact of Mn on mitochondria and autophagy flux. We started with in vitro models because they were more amenable to genetic and pharmacological manipulations. We performed time-course and dose-response cytotoxicity studies by exposing immortalized rat dopaminergic neuronal N27 cells, and HeLa autophagy reporter cells to increasing concentrations of MnCl_2_ up to 2,000 µM. Cell viability and Lethal Concentration-50 (LC_50_) values were calculated after 24 and 48 h of MnCl_2_ treatment. Mn decreased cell viability in both cell models (Fig. 1A and B) with LC_50_ values of 323.7 ± 8.7 µM (24 h) and 314.5 ± 15.8 µM (48 h) for HeLa cells, 260.6 ± 7.5 µM (24 h) and 186.9 ± 2.5 µM (48 h) for N27 cells. As compared to HeLa, neuronal N27 cells were more sensitive to Mn toxicity. Next, using autophagy HeLa reporter cells with stable expression of mRFP-GFP-LC3 (Fig. 1C) to monitor autophagy flux [49], we performed dose-response (15.6–250 µM) studies to determine the effects of Mn on autophagy (Fig. 1D and E). After Mn treatment for 24 h, the number of autophagosomes (green) increased, in a dose-dependent manner, while autolysosomes (red only puncta) markedly decreased compared to control cells. These results are indicative of a blockade in autophagy flux. At our lowest tested Mn concentration 15.6, autophagy flux was already impaired, with maximal inhibition at 125 µM.

Next, we investigated whether Mn impaired the early or late stage of autophagy flux. First, we assessed the late stage by treating HeLa autophagy reporter cells in the presence or absence of MnCl_2_, Chloroquine (CQ) or a combination of both. CQ is a lysosome inhibitor that interferes with the function and fusion of lysosomes with autophagosomes [50]. We hypothesized that since CQ blocks downstream autophagy, in the scenario where Mn predominantly blocks early stage of autophagy (induction or autophagosome function), more autophagy blockade would occur due to a combination of blocking autophagy flux at two separate stages. As seen in Fig. 1F and G, CQ blocked autophagy flux in a dose-dependent manner (50 and 100 µM). When combined with CQ 50 µM, a concentration that did not maximally inhibit autophagy, Mn at the concentration of 125 µM did not further impair autophagy – suggesting that Mn does not inhibit autophagy at an early stage, and as compared to CQ, it is a weaker inhibitor at the late stage. As a complementary approach, we further validated the effects of Mn at the late autophagy stage by assessing lysosomal acidification in dopaminergic N27 neuronal cells. Using the ratiometric probe LysoSensor Yellow/Blue. The ratio of emissions at 521/452 nm was calculated to monitor the changes of lysosomal pH. A reduced ratio indicates an increase in pH. As shown in Fig. 1H, Mn reduced lysosomal acidification in a dose-dependent manner, indicating Mn impairs lysosomal function. We then assessed the levels of Atg5, which is an integral part of the Atg5-Atg12-Atg1L16 complex, a marker for autophagosomes, in N27 cells treated with Mn 125 µM, with or without CQ 50 µM. As seen in the immunoblotting data (Fig. 1I and J), a combination of CQ and Mn did not alter the levels of Atg5 - further supporting the lack of effects of Mn at upstream autophagy. Together, these data demonstrate that at low concentrations, Mn inhibits autophagy flux at the lysosomal level.

### Mn does not affect mitochondria at the lower concentrations that impair autophagy

**Fig. 2:**
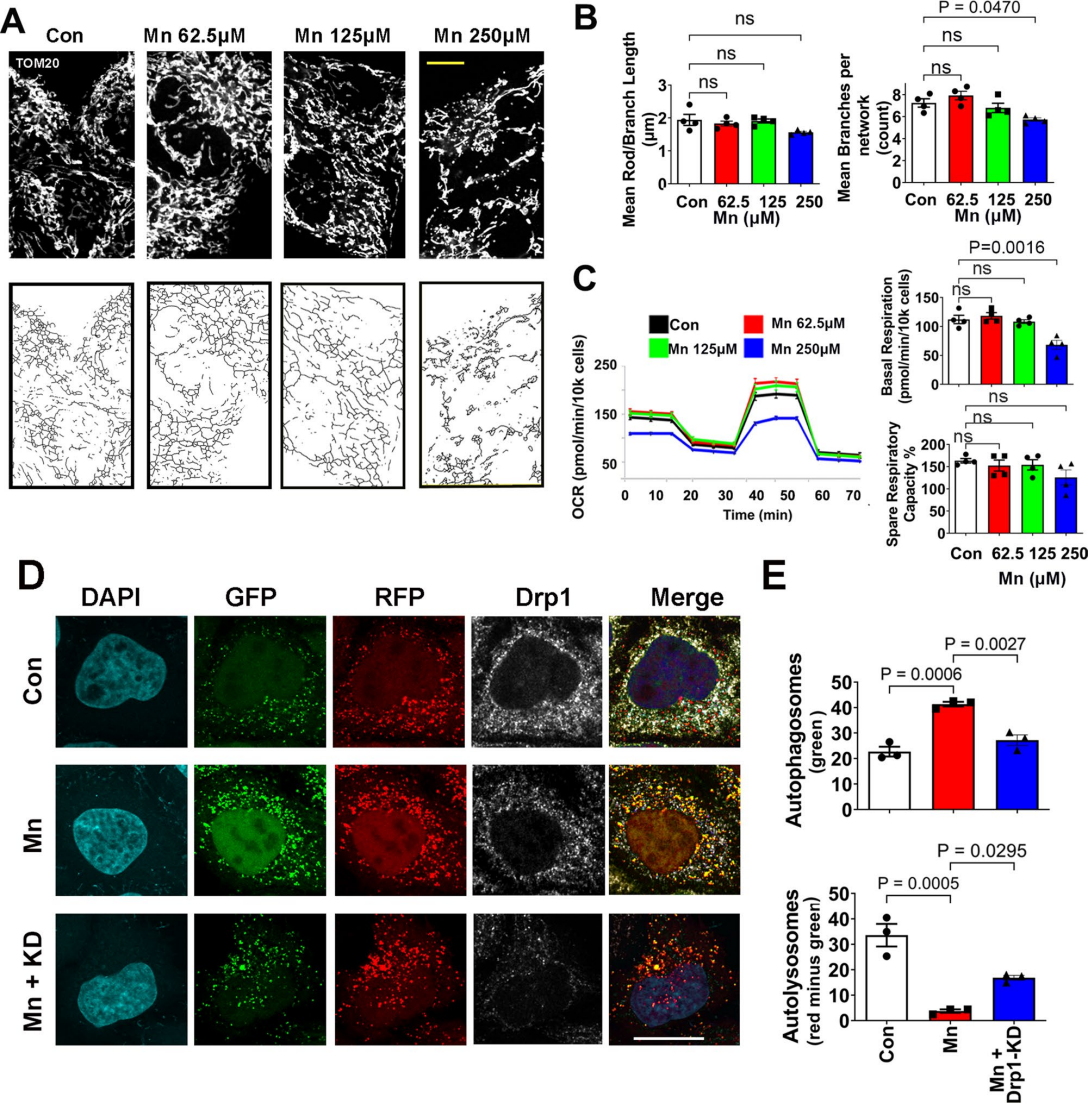
Low Mn concentrations do not affect mitochondria and inhibition of Drp1 attenuates its blockade on autophagy. HeLa autophagy reporter cells were treated with vehicle (DMEM media), and varying concentrations of Mn for 24 h. (**A**) Representative confocal images of mitochondrial morphology after TOM20 immunostaining (upper panels, scale bar 20 μm), and then skeletonized (lower panels) for subsequent analysis of mitochondrial morphology. (**B**) The MiNA plugin of Fiji ImageJ was used to quantified mitochondrial morphology/network. Data represent mean ± SEM (*n* = 4 independent experiments with 30–40 cells per group quantified per experiment), analyzed by one-way ANOVA, followed by Tukey’s post hoc test. Mn did not induce a significant change in rod or branch length (*P* = 0.8500 for Mn 62.5 µM, *P* = 0.9951 for Mn 125 µM and *P* = 0.0739 for Mn 250 µM). At 250 µM, a modest reduction in the number of branches per network (*P* = 0.0470) was detectable, but not at the lower doses (*P* = 0.5492, *P* = 0.8068 for 62.5 and 125 µM respectively). (**C**), Mitochondrial respiration was assessed by measuring OCR using the XF^e^96 Extracellular Flux Analyzer. Data represent mean ± SEM, *n* = 4 independent experiments with 8 replicates per experiment. (**D**) HeLa autophagy reporter cells were transfected with siRNA-Drp1 for 24 h to knockdown (KD) Drp1, and then treated with Mn (62.5 µM) for another 24 h, followed by immunostaining for Drp1. (**E**) Images were captured and the number of autophagosomes and autolysosomes were quantified as described in Fig. 1. Data represent mean ± SEM (*n* = 3 independent experiments with approximately 30 cells per experiment) and groups were compared by using Kruskal-Wallis ANOVA test with Dunn’s post-hoc test. Scale bar = 20 μm

To investigate whether at the low concentrations that Mn impairs autophagy flux (as seen in Fig. 1) mitochondria would also be impacted, we assessed mitochondrial morphology and function in the same cell model. To this end, a dose-response study of Mn (62.5 µM-250 µM for 24 h) was performed in HeLa autophagy reporter cells. Mitochondrial morphology was visualized using TOM20 immunostaining, followed by confocal microscopy (Fig. 2A). To quantify mitochondrial morphology and network more objectively, we used Mitochondrial Network Analysis (MiNA) of Fiji Image J. As shown in Fig. 2B, Mn did not cause mitochondrial fragmentation until it reaches 250 µM, a concentration that far exceeded the inhibitory effect of Mn on autophagy flux and caused˜ 36% cell death. To further validate the effect of Mn on mitochondria, we measured mitochondrial respiration using the Seahorse XF^e^96 extracellular flux analyzer. This experiment allowed us to determine the underlying mitochondrial dysfunction by serial injection of electron transport chain (ETC) modulators to assess different states of respiration and the proton circuit, thereby enabling the detection of subtle changes in the ETC before major disfunction occurs [51]. Consistent with the morphological data, mitochondrial function was not impaired by Mn unless it reached 250 µM (Fig. 2C). We also confirmed that at 125 µM, Mn did not affect mitochondrial membrane potential using TMRM (Supplementary Fig. S1). Together, these morphological and functional studies of mitochondria indicate that autophagy flux is more vulnerable than mitochondria to Mn toxicity in this cell model.

### Drp1 inhibition attenuates impairted autophagy induced by Mn in HeLa autophagy reporter cells

To evaluate the effects of blocking Drp1 on autophagy flux, HeLa autophagy reporter cells were transfected with siRNA-Drp1 for 24 h and then treated with or without MnCl_2_ for another 24 h (Fig. 2D). Approximately 70–80% of Drp1 knockdown efficiency was achieved in HeLa and N27 cells used in this study, whereas scramble-siRNA did not affect Drp1 levels (Supplementary Fig. S2), consistent with our previous validation [13]. This level of reduced Drp1 did not impair mitochondrial function (Supplementary Fig. S3), morphology, or autophagy [13]. However, as shown in Fig. 2E, Drp1 knockdown significantly attenuated autophagy blockade induced by Mn as evidenced by a reduction in the number of autophagosomes (from 41.35 ± 0.91 to 27.21 ± 2.08) and increased autolysosomes (from 3.73 ± 0.66 to 16.84 ± 0.94), respectively. In combination, these data indicate that reduced Drp1 improves autophagy flux in a cell model with impaired autophagy independent of abnormal mitochondrial function and morphology.

### Drp1 inhibition attenuates impaired autophagy and protein aggregation induced by Mn in dopaminergic neuronal cells

**Fig. 3:**
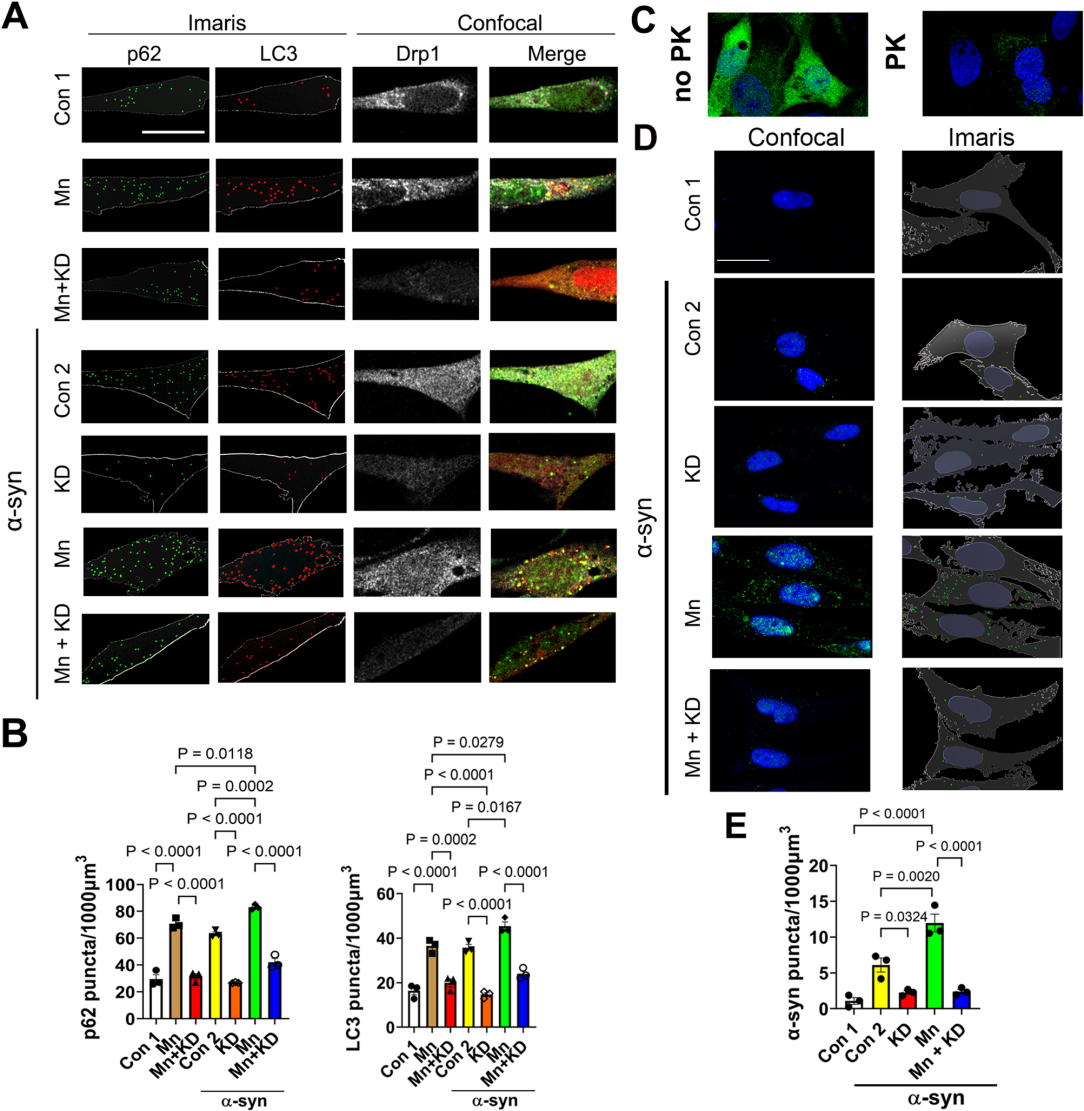
Drp1 inhibition protects against impaired autophagy and protein aggregation induced by Mn in N27 dopaminergic neuronal cells. Stable N27 cells expressing inducible human wild-type α-synuclein were co-transfected with siRNA-Drp1 (KD) and LC3-mcherry (due to low endogenous LC3 levels in this cell type) for 24 h. (**A**) Cells were then treated for another 24 h with MnCl2 (125 µM) or vehicle controls (Con 1 & Con 2), in the presence or absence of PonA (20 µM) to induce α-synuclein expression. (**B**), Following confocal imaging, the number of green and red vesicles, representing respectively p62 and LC3 puncta was quantified using Imaris image analysis software. Data represent mean ± SEM (*n* = 3 independent experiments, with at least 15 cells for each condition). Kruskal-Wallis ANOVA was used to compare between groups with Dunn’s post-hoc test. Scale bar 20 μm. (**C**), Representative confocal images showing total α-synuclein immunostaining (green) with or without Proteinase-K (PK) treatment. (**D**), N27 cells were transfected with siRNA-Drp1 (10nM) for 24 h, followed by α-synuclein overexpression induction (PonA20 µM), with and without Mn (125 µM) for 48 h. After post-fixation, cells were incubated with PK and then incubated with an antibody that detects α-synuclein (Millipore, AB5038). (**E**) Imaris was used to quantify PK-resistant α-synuclein-positive puncta. The number of aggregates was normalized to cytoplasmic volume. Data are shown as mean ± SEM (*n* = 3 independent experiments, with a minimum of 40 cells per group). Scale bar 20 μm

To further corroborate the observations in HeLa cells as shown in Figs. 1 and 2, we used the N27 rat dopaminergic neuronal cells with ecdysone-inducible expression of human wild-type α-synuclein [13]. First, we assessed the effects of Mn on autophagy flux and whether reduced Drp1 levels would also be protective in these neuronal cells by quantifying the number of LC3 and p62 immuno-positive puncta per using Imaris, a 3-D rendering software. As shown in Fig. 3A and B, Mn (125 µM) alone increased both LC3 (36.46 ± 1.77) and p62 (70.70 ± 2.27) puncta compared to vehicle cells (LC3: 16.36 ± -1.80; p62: 29.60 ± 3.29), indicating a blockade in the autophagy pathway. In contrast, inhibition of Drp1 attenuated the accumulation of LC3 (19.96 ± 1.79) and p62 (31.41 ± 2.25) puncta induced by Mn. These results are consistent with those in HeLa cells (Fig. 2). In addition, upon co-treatment of ponasterone A (ponA, an ecdysone analog used to induce α-synuclein expression) and Mn, the number of LC3 (45.38 ± 1.97) and p62 (83.12 ± 1.15) puncta increased to levels that were statistically different to cells exposed to PonA (LC3, 35.74 ± 1.478; p62, 63.19 ± 1.51) or Mn alone (LC3, 36.46 ± 1.77; p62 70.70 ± 2.27). These results indicate that α-synuclein alone blocked autophagy, as did Mn, however a more pronounced inhibition was observed when α-synuclein was combined with Mn. Drp1 knockdown using siRNA significantly reduced LC3 and p62 puncta in both PonA induced cells (LC3, 14.69 ± 0.90; p62, 26.87 ± 0.25) and cells co-treated with PonA and Mn (LC3, 24.02 ± 1.37; p62,42.14 ± 2.82.

Given that autophagy is a primary pathway by which misfolded/aggregated α-synuclein is removed, we assessed the effects of Mn and a partial Drp-1 knockdown on the levels of α-synuclein in this cell model using Imaris. These cells accumulate proteinase-K resistance α-synuclein after two days of induction by PonA (vehicle control: 1.08 ± 0.47; PonA: 6.10 ± 0.98) (Fig. 3C and D). However, Mn (125 µM) significantly increased the number of these aggregates (11.96 ± 1.25). Knocking down Drp1 drastically reduced such protein aggregation (2.39 ± 0.29). Overall, these data suggest that autophagy dysfunction and α-synuclein aggregation induced by Mn can be ameliorated by Drp1 inhibition.

### Mn does not perturb mitochondrial morphology and function at low concentrations in dopaminergic neuronal cells

**Fig. 4:**
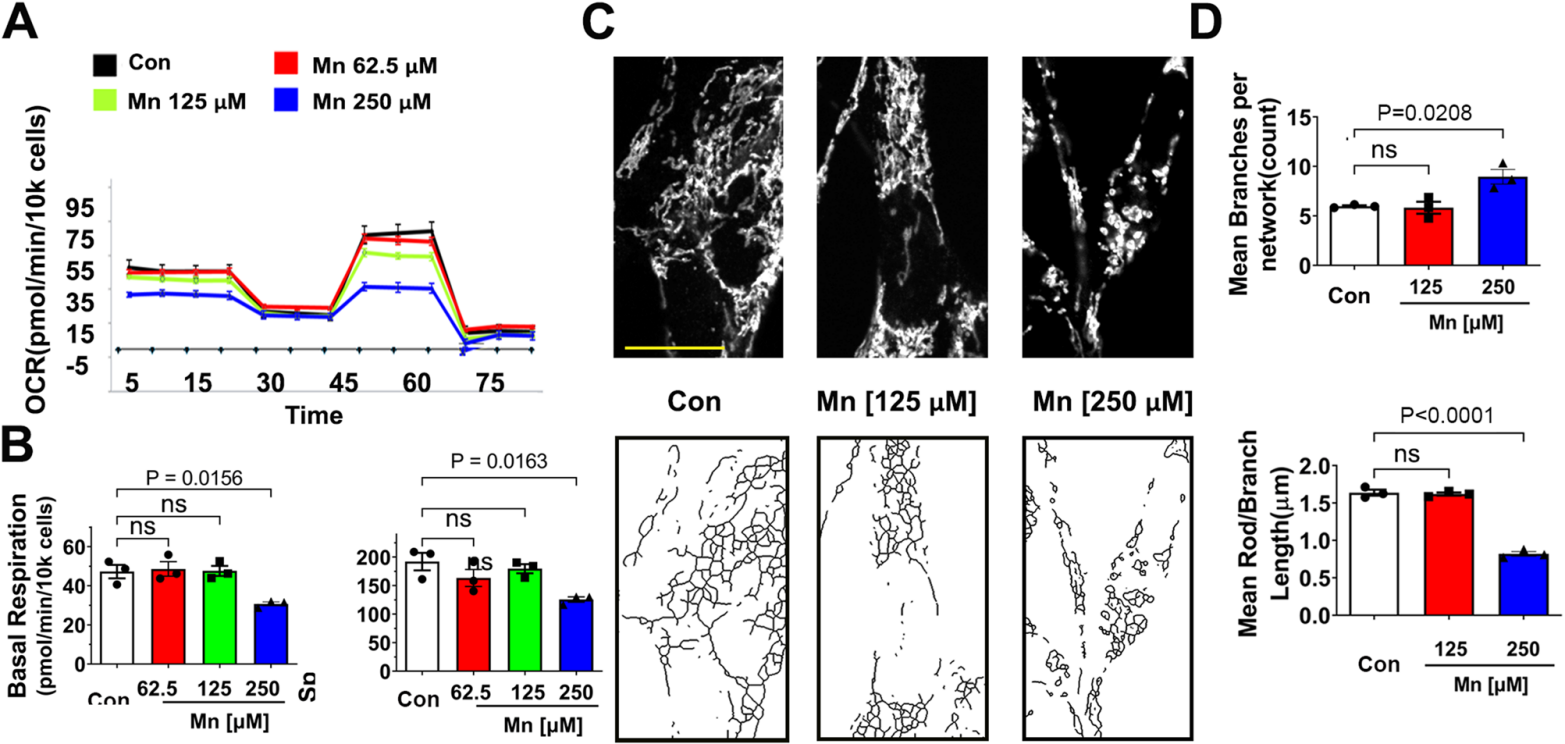
Mn does not affect mitochondria at low concentrations in N27 dopaminergic neuronal cells. (**A,B**) Mitochondrial respiration was assessed by measuring OCR using the XF^e^96 Extracellular Flux Analyzer. At 62.5 and 125 µM, Mn did not impair basal respiration (*P* = 0.9859, *P* = 0.9997 respectively) or spare respiratory capacity (*P* = 0.3693, *P* = 0.8727 respectively) Data represent mean ± SEM, *n* = 3 independent experiments (**C**) N27 neuronal cells were treated with 125 µM or 250 µM of MnCl2 or vehicle control (RPMI media) for 24 h. Representative confocal images of mitochondrial morphology were captured after TOM20 immunostaining (upper panels), then skeletonized (lower panels) for subsequent analysis. Scale bar 20 μm. (**D**). Various parameters of mitochondrial morphology were quantified using Fiji MiNA plugin. No significant changes in branch length (*P* = 0.9082) or the number of branches per network (*P* = 0.9806) was detectable in 125 µM Mn treatment. Data represents mean ± SEM (*n* = 3 independent experiments with 30–40 cells per experiment), analyzed by Kruskal-Wallis ANOVA test

In the autophagy reporter HeLa cells, we observed that autophagy was inhibited by Mn at concentrations that did not affect mitochondria (Fig. 2). We asked whether Mn would also have the same effects in neuronal cells. Using N27 dopaminergic neuronal cells, we performed a dose-response study of Mn (62.5–250 µM) and quantified mitochondrial morphology and function. As shown in Fig. 4, Mn did not cause abnormal mitochondrial function and morphology up to 125 µM, a concentration that impaired autophagy flux in this cell type. At 250 µM, which caused˜ 40% cell death, Mn did induce mitochondrial fragmentation and impaired mitochondrial respiration.

### Transcriptomic analysis reveals Mn impairs autophagy but not mitochondrial pathways in vivo

**Fig. 5:**
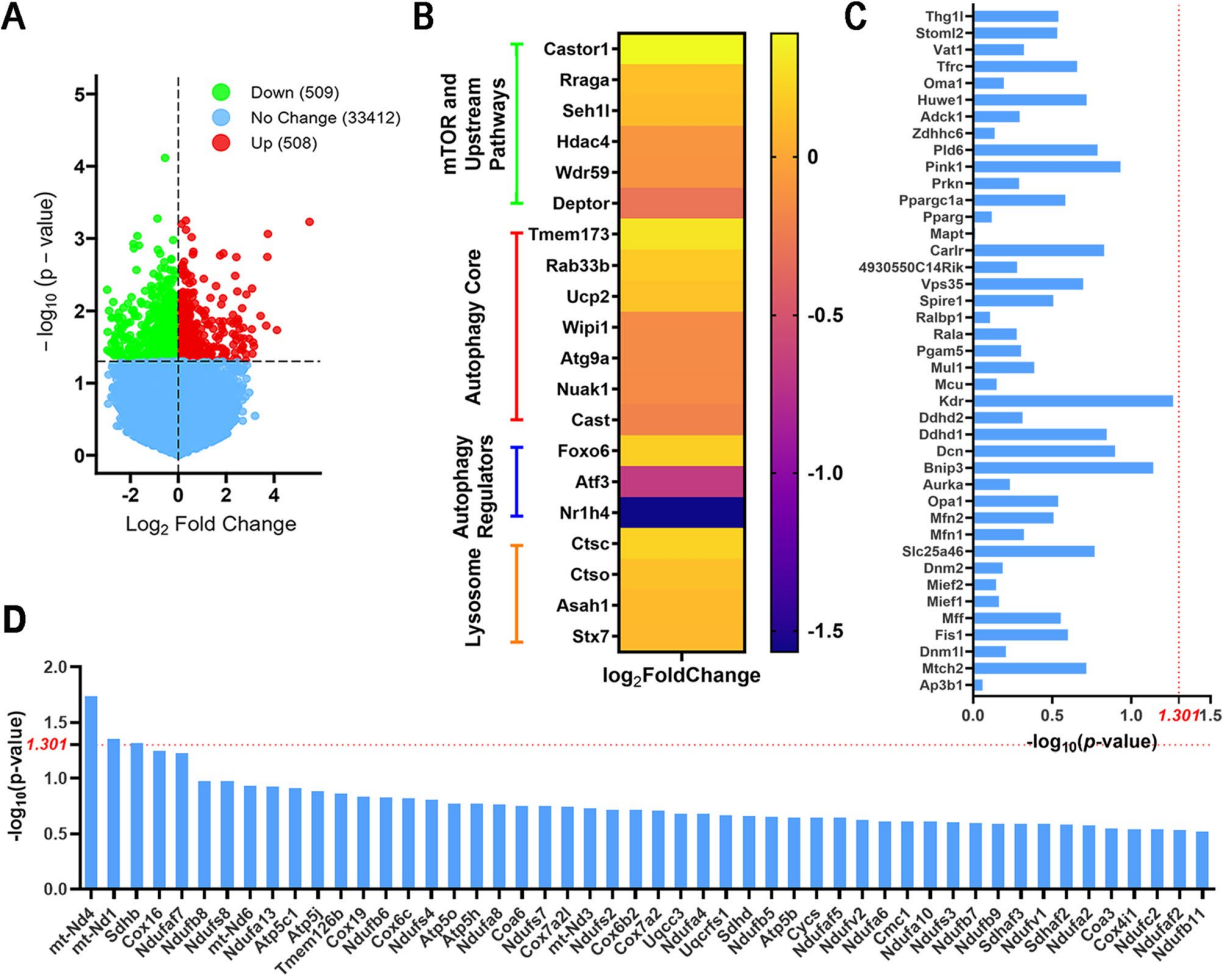
RNAseq reveals Mn impairs autophagy but not mitochondrial pathways in vivo. C57/BL6 mice (3–4-month-old) were orally gavaged with MnCl_2_ 4H_2_O (15 mg/kg/day) or water control for 30 consecutive days before harvesting (*n* = 8 for each group). Total RNA was extracted from the ventral midbrain and processed for RNAseq. (**A**) Volcano plot showing the number of differentially expressed genes between the Mn-treated mice and their control littermates (> one-fold change, *p* < 0.05). (**B**) Heat map showing differential expressed genes (DEGs) of the autophagy pathways between the Mn-treated mice and the control mice. (**C**) Genes related to mitochondrial dynamics were not significantly affected after Mn exposure [-log_10_(p-value) < 1.301]. (**D**) Genes involved in mitochondrial function show 97.7% (128/131 genes) were not significantly affected in the Mn-treated mice compared to the control group [-log_10_(p-value) < 1.301]. For brevity, only the top 50 genes are shown here. Additional genes are included in the Supplementary file 1

As demonstrated in our in vitro data using complementary approaches (Figs. 1, 2, 3 and 4), when the concentration of Mn was titrated, it was clear that the inhibitory effects of Mn on autophagy and mitochondria were dose-dependent. To investigate the effects of Mn on autophagy and mitochondria in vivo, we started with an unbiased approach by performing RNAseq to identify changes in the transcriptomic profile in the ventral middle brain of the C57/BL6 mice treated orally with either MnCl_2_ 4H_2_O (15 mg/kg/day, equivalent to 4.2 mg absolute Mn/kg/day) or water control once daily for 30 consecutive days [43]. This low dose and chronic Mn regimen was selected for its lack of neurotoxicity by itself but sufficient to induce dopaminergic neurodegeneration, protein aggregation and exosome release in mice when combined with viral-mediated α-synuclein overexpression [43]. From the RNAseq analysis, we found 508 genes were up-regulated and 509 genes were down-regulated in response to Mn exposure (*p* < 0.05) (Fig. 5A). Among those differentially expressed genes (DEGs), we further profiled the ones that were specifically involved in the autophagy pathways using “Autophagy Transcription Gene Toolbox” [44]. Results revealed that the four subsets of autophagy genes that were significantly dysregulated by Mn treatment belong to “mTOR and upstream pathways”, “Autophagy Core”, “Autophagy Regulators” and “Lysosome” (*p* < 0.05) (Fig. 5b). No sex differences were observed within the Mn-treated mice as well as the control mice; therefore, we combined the results (*n* = 4 females and *n* = 4 males per group) for these autophagy associated DEGs (Supplementary File 1). Because some of these genes have either inhibitory or activating effects on autophagy function, it is not feasible to conclude whether or how these alterations would ultimately affect autophagy. Therefore, we used KEGG pathway analysis to examine whether the autophagy pathways (Supplementary Fig. S5) would be enriched either in “up-regulated” or “down-regulated” pathways after exposure to Mn. The results showed a significant (padj = 0.0128) downregulation in the ventral midbrain of the Mn-treated mice as compared to the vehicle control group (Supplementary File 1, Supplementary Fig. S5). These results indicate the impairment of autophagy upon Mn exposure in vivo.

To assess the effects of Mn on mitochondria at the transcriptional level in these animals, genes that are associated with mitochondrial dynamics and mitochondrial function as well as mitophagy were compared between the Mn-treated mice and control mice. As shown in Fig. 5C, no genes involved in mitochondrial dynamics were significantly affected by Mn treatment, and 97.7% (128/131) genes responsible for mitochondrial function were not affected (Fig. 5D, Supplementary File 1). The three significantly affected genes were *mt-Nd4* (*p* = 0.01845, Log2Fold Change = 0.1405), *mt-Nd1* (*p* = 0.04453, Log2Fold Change = 0.1382) and *Sdhb* (*p* = 0.04861, Log2Fold Change = 0.1841), all of which are subunits of mitochondrial complex 1 and slightly upregulated after exposure to Mn (Fig. 5D, Supplementary File 1). In addition, 96.3% (77/80) genes that are involved in the mitophagy pathway were not affected by the Mn treatment after profiling using “Autophagy Transcription Gene Toolbox”. The three DEGs were all “Negative Regulators of Mitophagy”, among which *Usp30* (*p* = 0.02746, Log2Fold Change = 0.1010) and *Tmem173* (*p* = 0.0402, Log2Fold Change = 0.2979) were upregulated, while *Siah3* (*p* = 0.0444, Log2Fold Change=-0.2060) was downregulated (Supplementary File 1). Further KEGG pathway analysis using “Mitophagy-animal (mmu04137)” demonstrated that mitophagy was not affected (padj = 0.9893) (Supplementary File 1). Cumulatively, these results indicate that the repeated low-dose Mn treatment impairs autophagy but not the pathways related to mitochondrial dynamics, mitochondrial function and mitophagy. These in vivo data further strengthen the in vitro results that autophagy flux is more vulnerable than mitochondria to Mn toxicity.

### Mn impairs autophagy flux in nigral dopaminergic neurons

**Fig. 6:**
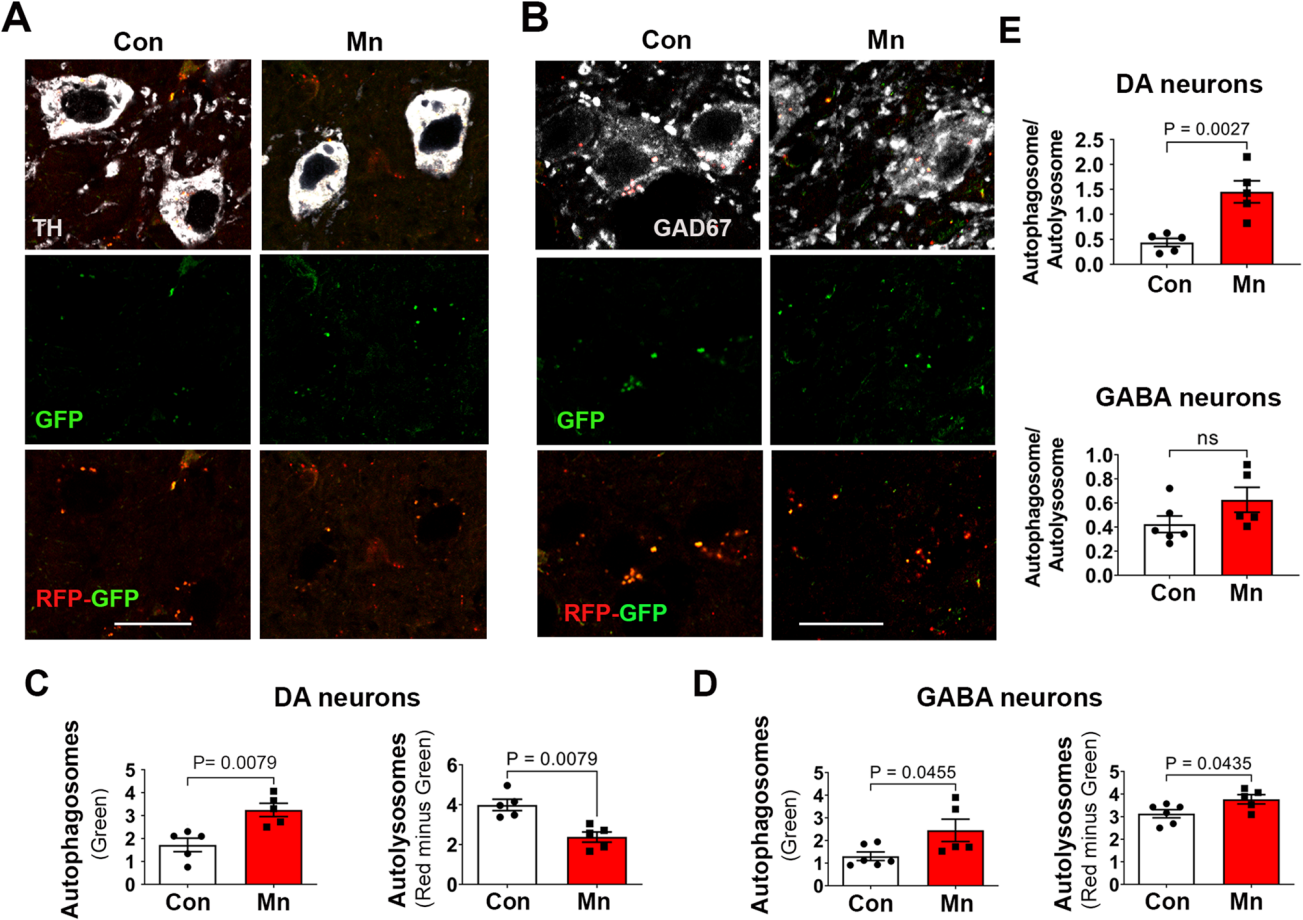
Selective autophagy impairment induced by Mn in the ventral midbrain. Autophagy reporter mice [C57BL/6-Tg(CAG-RFP/EGFP/Map1lc3b)1Hill/J] were treated with MnCl_2_ 4H_2_O (15 mg/kg/day) or water through oral gavage once daily for 30 consecutive days, then perfused for immunostaining and confocal imaging of DA neurons (**A**), tyrosine hydroxylase-positive and GABA neurons (**B**), GAD67-positive. Autophagic vesicles were quantified for autophagosome and autolysosome as described in Fig. 1 and normalized as per 100 µm^2^ for DA neurons (**C**) and GABA neurons (**D**) The conversion rate of autophagosome to autolysosome was calculated by expressing the ratio of these two types of vesicles (**E**). Significant difference between control and Mn treatment group was not observed in GABA neurons (*P* = 0.1257). Data represent Mean ± SEM, *n* = 5 per group, with approximately 40–50 neurons analyzed per animal. The number of autophagosomes, autolysosomes, and their ratio were compared using an independent t-test

Although our transcriptomic data show impaired autophagy pathways, we further validated autophagy flux at the cellular protein levels using transgenic autophagy reporter mice that express ubiquitously a tandem RFP-EGFP-LC3 fusion protein [52], the same strategy used to generate the stable autophagy reporter cells used in our in vitro studies (Fig. 1). These mutant mice were treated with the same oral MnCl_2_ regimen as in the RNAseq study. Given the potential role of Mn as a risk factor in PD, we assessed autophagy flux in dopamine (DA) neurons in the substantia nigra *pars compacta* (SNpc, Fig. 6A). We also included GABA neurons in the substantia nigra *pars reticulata* (SNpr) as control (Fig. 6B). Using confocal microscopy, we captured the red and green fluorescent puncta and quantified the number of autophagosomes and autolysosomes. As seen in Fig. 6C, Mn impaired autophagy in tyrosine hydroxylase (TH, a phenotypic marker for DA neurons) positive neurons as evidenced by an increase in autophagosomes and a decrease in autolysosome. In contrast, Mn did not impair autophagy flux in GAD67 positive GABA neurons (Fig. 6D). Although the number of increased autophagosomes was marginally significant, there was no reduction in the autolysosome levels. In fact, there was a marginal, but statistically significant, increase in autolysosomes. Next, we evaluated the conversion rate of autophagosome to autolysosome by calculating the ratio of these two vesicles (Fig. 6E). Mn decreased this conversion rate in DA neurons, suggesting the impairment is mostly impacted by downstream of lysosomal fusion and degradation, rather than the upstream of autophagy induction. This observation is consistent with the in vitro data (Fig. 1F-I).

### Generation and characterization of Drp1-deficient mice

**Fig. 7:**
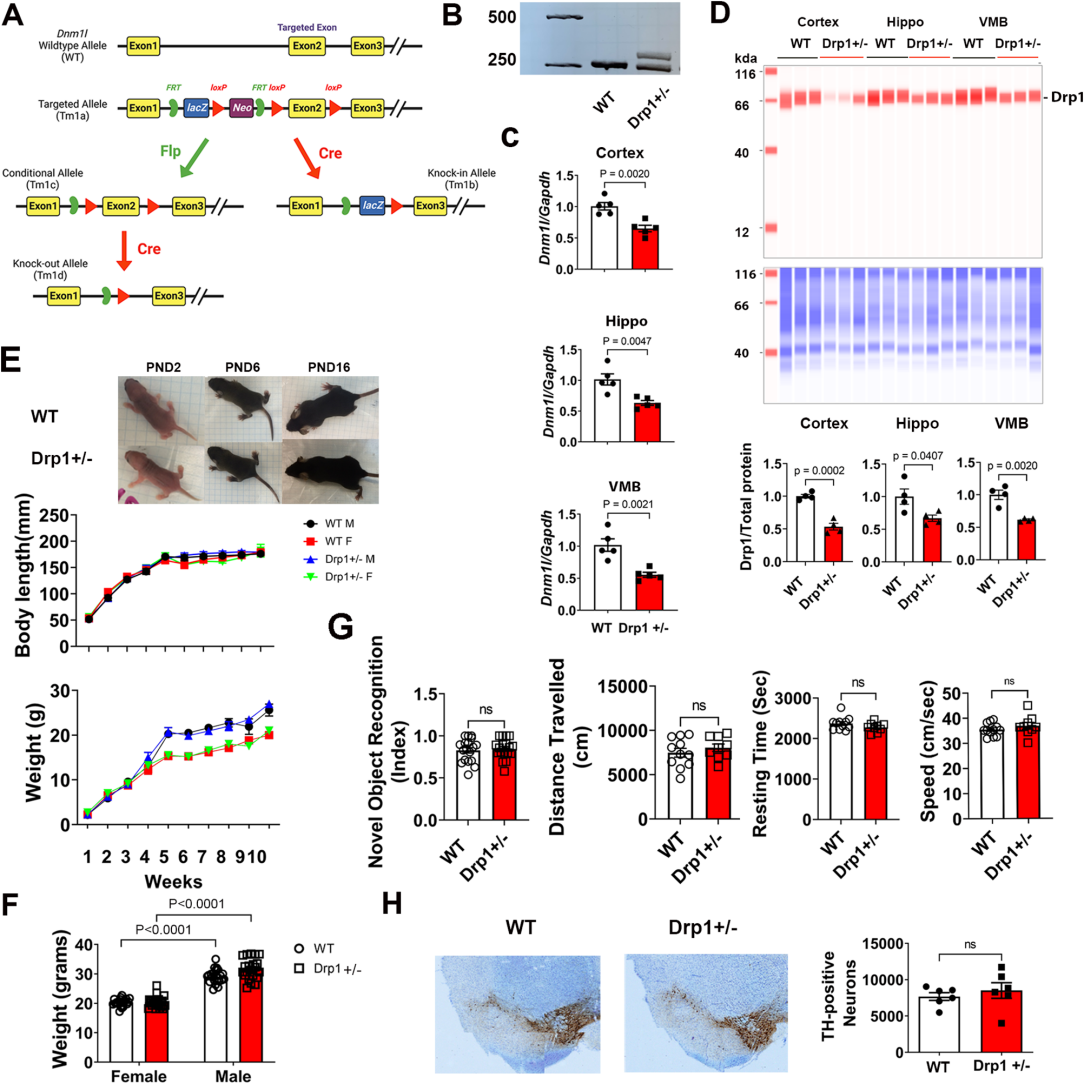
Generation and characterization of Drp1 heterozygous global knockout (Drp1^+/−^) mice. (**A**) Schematic diagram illustrating the targeting strategy for generating different types of Drp1-deficient mice using the “knockout-first” technology. The KO-first allele is flexible and can produce reporter knockouts, conditional knockouts, and null alleles following exposure to site-specific recombinases Cre and Flp to delete Exon 2 of the *Dnm1l* gene. (**B**) representative genotyping results of Drp1^+/−^ versus WT controls. (**C**) qPCR and (**D**) immunoblotting confirmed the reduction of mRNA and protein levels of Drp1 in the Drp1^+/−^ mice in different brain regions (cortex, hippocampus, and ventral midbrain). (**E, F**) Weekly measurement showed no differences in body weight and length between Drp1^+/−^ and WT littermates during the developmental stage, *n* = 66 WT (44M & 22 F), and 63 Drp1^+/−^ (33M & 30 F). (**G**) No differences in locomotor activity (*P* = 0.4066 for distance travelled, *P* = 0.1671 for resting time, *P* = 0.3282 for speed, *n* = 11 WT, *n* = 9 Drp1^+/−^), as well as learning and memory test (*P* = 0.6736, *n* = 18/group) between genotypes. (**H**) Stereological counting of DA neurons in the SNpc show no differences between genotypes (data represent Mean ± SEM, *n* = 6). Statistical comparison was performed using either independent t-test or Two-Way ANOVA.

To evaluate the protective effects of a partial Drp1 deficiency against Mn-induced autophagy in the brain, we generated Drp1-knockout (KO) mice using targeting strategies as illustrated in Fig. 7A. We maintained heterozygous Drp1-KO (Drp1^+/−^) and their wild-type (WT) littermate (Fig. 7B) because homozygous disruption of *Dnm1l*, the gene encoding Drp1, is embryonically lethal [53, 54], and a conditional complete deletion of *Dnm1l* causes dopaminergic cell death in adult mice [55]. Quantitative analysis using qPCR (Fig. 7C) and immunoblotting (Fig. 7D) in multiple brain regions (cortex, hippocampus, and ventral midbrain) confirmed an approximately 50% reduction of Drp1 levels in Fig. 7 the mutant mice. Physically (Fig. 7E and F) and behaviorally (Fig. 7G), there were no differences between Drp1^+/−^ and WT mice. Stereological cell counting shows a similar number of DA neurons in the SNpc (Fig. 7H). Together, these data indicate that a partial reduction of Drp1 in the heterozygous mice does not negatively impact animal development or DA neuron viability.

### Drp1^+/−^ mice is protective against autophagy impairment induced by Mn

**Fig. 8:**
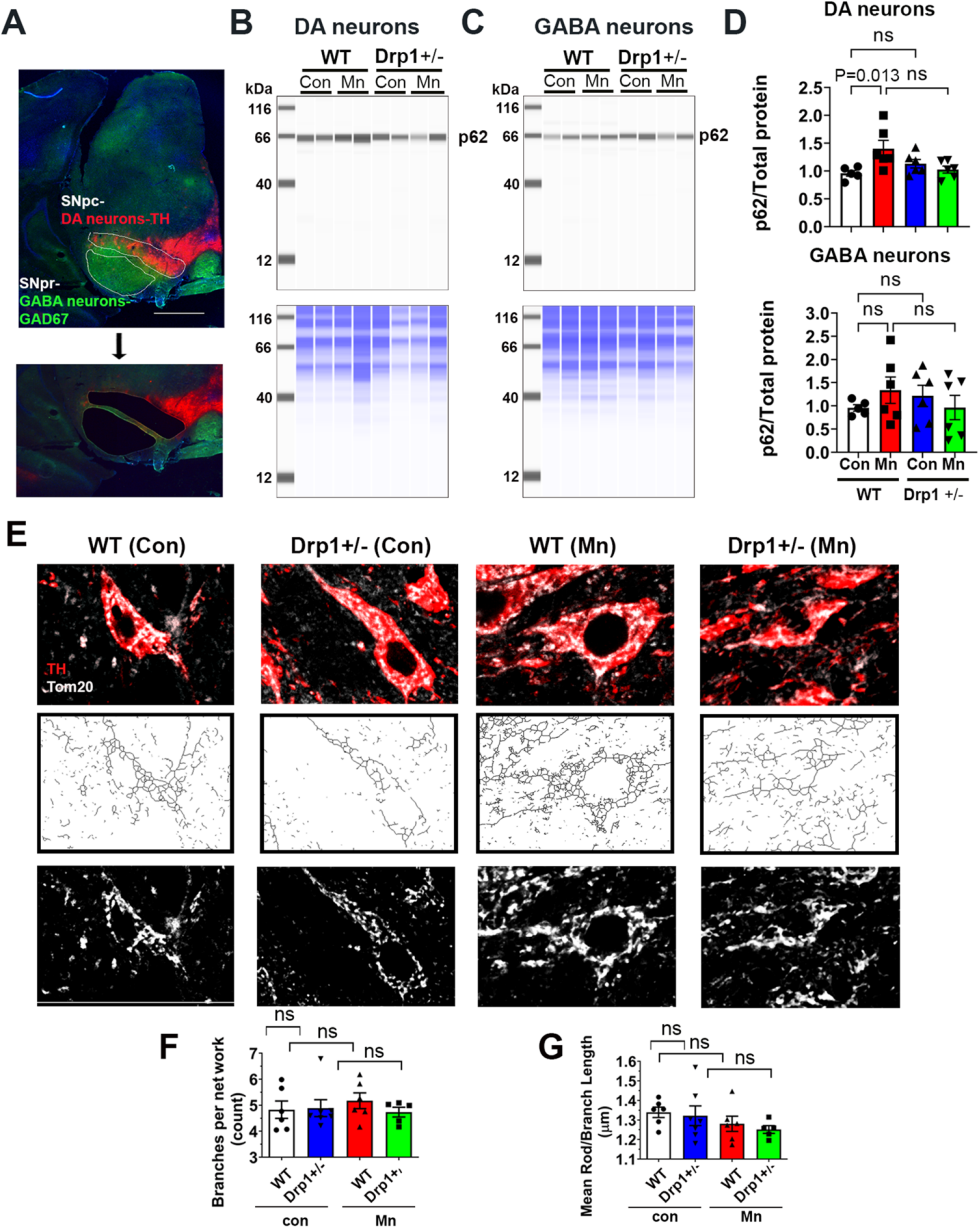
Drp1^+/-^ mice is protective against autophagy impairment induced by Mn. (**A**) Representative images of the coronal mouse midbrain section (20 μm) co-immunostained for DA neurons (TH, red) in the SNpc and GABA neurons (GAD67, green) in the SNpr. Both of these brain regions were removed by laser microdissection for immunoblotting of p62 (top panels) in DA neurons (**B**) and GABA neurons (**C**). Total proteins per lane (bottom panels) were used as loading control. (**D**) Quantified levels of p62 were significantly increased in DA neurons (*P* = 0.0013), but not in the GABA neurons (*P* = 0.5457), of the Mn-treated WT mice. Mn did not significantly increase p62 in DA neurons of the Drp1^+/−^ mice (*P* = 0.8660) No significant baseline level differences between the two genotypes were observed (*P* = 0.5664 for TH neurons and *P* = 0.7675 for GABA neurons. Data represent mean ± SEM, *n* = 5 for WT control, *n* = 6 for other groups), two-way ANOVA followed by Tukey post-hoc test. (**E**) Representative confocal images of mitochondrial morphology of DA neurons after TOM20 immunostaining (upper panels), then skeletonized (middle panels) for subsequent analysis. Scale bar 20 μm. (**F, G**) Various parameters of mitochondrial morphology were quantified using Fiji MiNA plugin. Data represents mean ± SEM (*n* = 6 mice per group), analyzed by one-way ANOVA, followed by Tukey post-hoc test

Based on the observation that Mn selectively impaired autophagy in nigral DA neurons but not their GABA counterparts (Fig. 6), we used laser capture microdissection (as illustrated in Fig. 8A) to specifically remove SNpc (rich in DA neurons) and SNpr (rich in GABA neurons) in Drp1^+/−^ mice and their WT littermates treated with oral MnCl_2_. With these tissues, we performed immunoblotting for p62 levels using Jess™ system (ProteinSimple, Inc). Mn significantly increased the p62 protein levels in the nigral DA neurons from WT mice but not in the Drp1^+/−^ mice. However, Mn treatment did not affect p62 protein levels in the nigral GABA neurons from either Drp1^+/−^ mice or WT littermates (Fig. 8B-D). These data also show that autophagy was not affected in the Drp1^+/−^ mice as compared to WT littermates under basal conditions.

To assess whether the mitochondrial network was also affected in DA neurons, we quantified mitochondrial morphology in these animals (Fig. 8E-G). No differences in the mitochondrial network were detectable between genotypes as well as between Mn treatment and vehicle control. Collectively, these results indicate that at a low chronic oral dose, Mn selectively impairs autophagy in nigral DA neurons which are protected by Drp1 inhibition. Furthermore, this protection is independent of the mitochondrial fission role of Drp1.

## Discussion

Mitochondrial dysfunction is a pathogenic mechanism in neurodegenerative diseases such as AD, PD, HD and ALS [5, 6]. For PD, the discovery of MPTP in the early 1980’s, and the subsequent discoveries of PINK1, parkin being involved in mitochondrial function and quality control [56], have put the spotlight on the significant role of mitochondria. The observations that PINK1 and parkin affect mitochondrial dynamics has expanded the interest beyond the electron transport chain to include mitochondrial fission, fusion, and movement, not just in PD but also in other neurodegenerative diseases such as AD, HD and ALS [5, 6]. Restoring the balance in mitochondrial fission and fusion is one potential common therapeutic approach for these diseases. Blocking Drp1 using both genetic and pharmacological approaches has been shown to be protective in multiple models of neurodegenerative diseases [5, 6]. In addition to improving mitochondrial function and morphology, one striking feature in some of these studies is the observations of reduced protein aggregation of α-synuclein [12, 13], huntingtin [16], and amyloid-beta [14, 15]. Although it is possible that the enhanced clearance of these aggregates is due to improved mitochondrial function, it is also possible that blocking Drp1 has a direct impact on improving autophagy to remove misfolded/aggregated proteins, or a combination of both mechanisms as discussed below.

Autophagy and mitochondria are functionally linked in a bidirectional manner [33]. Autophagy controls mitochondrial quality and number by selectively removing depolarized and damaged mitochondria through a process known as mitophagy. PINK1 and Parkin, the two proteins involved in autosomal recessive PD play a critical role in mitophagy [57]. On the other hand, mitochondria can also control autophagy function. It has been demonstrated that the activity of autophagy is highly dependent on the metabolic state of mitochondria [58, 59]. For this reason, to evaluate whether Drp1 inhibition improves autophagy flux independent of improving mitochondrial function, it is critical to have a model without impaired mitochondria since blocking Drp1 itself may improve mitochondrial function, which in turn, improves autophagy. Current genetic models with gene-products linked to neurodegenerative diseases such as PD, AD, HD and ALS have been reported to impair mitochondrial function-either directly or indirectly [60].

To address the critical question of whether a partial Drp1 inhibition would improve autophagy flux independent of mitochondria, we searched for a neurological disease-relevant model that impairs autophagy without affecting mitochondria. We discovered that at low concentrations, Mn met these criteria. Using two cell culture models (stable autophagy HeLa reporter cells and N27 neuronal cells), we performed dose-response studies and revealed that there is a threshold (250 µM) that separated the effects of Mn on autophagy flux and mitochondrial function and morphology. Such threshold could be due to cytotoxic effects of Mn at this concentration as shown in our cell viability study and most likely, also cell type dependent. For example, in primary astrocytes, Mn inhibits both mitochondria and autophagy at 100 µM [32, 61]. These observations are consistent with the ability of astrocyte to concentrate Mn up to 50-fold greater than those in neurons through their expression of the transferrin receptors and divalent metal transporter [62]. It is likely that Mn also has a threshold effect on autophagy and mitochondria in primary astrocytes.

To strengthen our in vitro data, we used two mouse models (transgenic autophagy reporter mice and wild-type C57Bl mice). We treated them with a chronic and low-dose of oral Mn regimen that was previously reported to increase α-synuclein aggregation and transmission *via* exosome [43]. With these in vivo studies, we discovered that Mn selectively impaired autophagy flux in the SNpc DA neurons but not the nearby GABA neurons in the SNpr. Although this observation is highly interesting, elucidating the mechanism of this selective vulnerability is beyond the scope of this study. However, these results are consistent with the fact that SNpc DA neurons are the most vulnerable cell type in PD. Although the precise mechanism(s) remain to be firmly established, decades of research has provided some insights [63]. For example, SNpc DA neurons have long, poorly myelinated axon with extensive arborization. This feature leads to greater bioenergetic demands and mitochondrial oxidant stress [64]. Another unusual feature of this cell type is that they are autonomous pacemakers with broad action potentials coupled with large oscillations in intracellular Ca^2+^, which is driven by a rather unique L-type Ca^2+^ channel (Ca_v_1.3) expressed in nigal DA neurons [65]. Such intrinsic high cytosolic Ca^2+^ levels in these DA neurons (36), when combined with α-synuclein and DA, contribute to their selective cell vulnerability [66]. SNpc DA neurons also have lower autophagic capacity [63], making them more sensitive to the impact of Mn on autophagy flux. Overall, our data are consistent with the following long-standing knowledge in the field: First, SNpc DA neurons are highly sensitive to insults. Second, the major target of Mn neurotoxicity is GABA neurons in the globus pallidus [67], not those in the nigra. This selectivity is intriguing and analogous to the observation that DA neurons in the SNpc are more sensitive than their counterparts in the ventral tegmental area.

Mitochondrial network was not affected by this Mn regimen, suggesting that at this low dose of Mn ($\sim$4.2 mg/kg/day), a threshold by which Mn affects mitochondrial function has not reached. Due to the selective impact of Mn on nigral DA neurons, it was challenging to obtain sufficient tissue samples to assess whether mitochondrial function was impaired in these neurons. This technical challenge represents a limitation of this in vivo study. However, we believe our extensive in vitro studies complement well with the in vivo experiments. Furthermore, RNAseq data confirmed autophagy pathways were impaired but not mitochondrial nor mitophagy related genes. In combination, both in vitro and in vivo data address the objective of this study by demonstrating that a partial Drp1 inhibition improves autophagy flux independent of the mitochondrial fission role of Drp1. The precise mechanism by which Drp1 inhibition improves late stage of autophagy flux requires more extensive investigation which is beyond the scope of this study.

After characterizing the effects of Mn on autophagy and mitochondria, we proceeded to evaluate the effects of a partial Drp1 inhibition on autophagy flux. Due to the concern of potential off-target effects of Drp1 inhibitors and to avoid the possibility of completely blocking Drp1 function, we decided to genetically remove a partial function of Drp1. For in vivo studies, we used our recently generated Drp1^+/−^ mice, which expressed approximately 50% less Drp1 protein as compared to their WT littermates. In cell culture models, our gene silencing approach using SMARTPool siGenome siRNA reduced about 70% of Drp1 protein, consistent with our previous report [13]. In both in vitro and in vivo models, we demonstrated that a partial inhibition of Drp1 protected autophagy flux impairment induced by Mn. Autophagy plays a critical role in removing most long-lived and aggregated-prone proteins, including α-synuclein [59, 68–70]. Therefore, a blockade of autophagy flux would increase the accumulation of α-synuclein. Consistent with this mechanism, our data showing that Mn increased the accumulation of proteinase-K resistant α-synuclein. This increase will create a feedback loop [71] since α-synuclein itself inhibits autophagy function [72]. As a compensatory response to reduce intracellular accumulation of α-synuclein when lysosomal function is blocked, exosome-mediated transfer release of α-synuclein is increased [73–75]. This observation is consistent with the interacting pathways between autophagy flux and amphisome-mediated exosome release [75]. When autophagy flux is blocked, the multi-vesicular body and amphisome pathways are more active to reduce intracellular α-synuclein levels, leading to more exosomal release [75]. Consistent with the inhibitory effects of Mn on autophagy in the present study, a previous study reported that Mn increased the release of exosomes containing oligomeric synuclein [43]. Thus, by improving autophagy function, Drp1 inhibition can reduce α-synuclein aggregation and spread.

Acute high exposure to Mn causes manganism. Because the globus pallidus is the primary brain region affected, these parkinsonian-like symptoms are not responsive to levodopa [20–22]. However, low-level long-term exposure to Mn may extend to the entire basal ganglia, including the substantia nigra, as demonstrated experimentally in mice [26, 28]. In human occupational studies, when exposed to welding fumes which contain Mn, welders are more likely to develop PD/parkinsonism [26, 76–78]. Consistently with these previous investigations, a recent study reported that serum exosomes of welders contain higher misfolded α-synuclein [43]. In addition to occupational exposure, Mn in the living environment is also a concern. A high prevalence of parkinsonism has been observed in the province of Brescia, Italy, where residents live in the vicinities of ferroalloys industries operating since the beginning of 1900 [25, 79]. In the same province, case-control studies have shown a relationship between PD/parkinsonism with metal exposure and α-synuclein polymorphism [79]. These studies indicate that Mn may be a risk factor for developing PD, especially when interacting with an individual’s genetic makeup [24, 80]. Such interactions have been demonstrated experimentally where Mn is combined with PD-linked genes such as *SNCA*, *parkin, DJ-1*, and *ATP13A2* [23, 26, 62, 81]. Because PD-linked proteins and Mn share pathogenic mechanisms such as mitochondrial dysfunction, neuroinflammation, oxidative stress, and autophagy impairment [43], it is not surprising that when combined, their neurotoxic effects are amplified. Our data showing that at low concentrations, Mn impairs autophagy and enhances α-synuclein aggregation, consistent with the proposal that this heavy metal may increase the risk of developing PD.

## Conclusions

The present study provides data demonstrating that at low concentrations, autophagy, not mitochondria, is the initial vulnerable target of low Mn exposure. This negative impact of Mn on autophagy flux is consistent with the observations of increased protein aggregation when Mn is combined with α-synuclein. We also provide evidence showing that a partial reduction in Drp1 levels improves autophagy flux independent of mitochondrial function. Based on the observations that a partial loss of Drp1 function reduces protein aggregation in experimental models of PD [12, 13], AD [14, 15], and HD [16], as well as a recent study reporting that abnormal levels of autophagy related genes in the transgenic Tau-P301L mice are significantly normalized when crossed with Drp1^+/−^ mice, the role of Drp1 in autophagy is not confined to Mn-induced neurotoxicity. Although the present study addresses the role of Drp1 in autophagy, it does not undermine the significant and well-established function of this protein in mitochondrial fission. In fact, our work highlights the central role of Drp1 as a linker between autophagy and mitochondria, consistent with the bidirectional regulation of these two pathways [33]. Given that impaired autophagy and mitochondrial dysfunction are two prominent features of neurodegenerative diseases, the neuroprotective effects of Drp1 inhibition mediated through improved autophagy flux (as shown in this study) and mitochondrial function (as reported in other studies) make this protein an even more attractive therapeutic target. Furthermore, although a complete Drp1 deletion is detrimental [53–55], our heterozygous Drp1-KO mice, as well as another similar model [82] exhibit normal life span, phenotype, autophagy/mitophagy, synaptic structure, as well as mitochondrial morphology and function. Therefore, a partial Drp1 loss of function appears to be safe and sufficient to confer neuroprotection mediated by its role in mitochondria and autophagy.

### Electronic supplementary material

Below is the link to the electronic supplementary material.

**Supplementary Material 1: Fig. S1.** Effects of 125 µM Mn treatment on mitochondrial membrane potential

**Supplementary Material 2: Fig. S2.** Efficiency of siRNA-mediated Drp1-KD in HeLa and N27 cells

**Supplementary Material 3: Fig. S3.** Effects of Drp1 knockdown on mitochondrial respiration

**Supplementary Material 4:** Supplementary file 1-RNAseq data

**Supplementary Material 5: Fig. S5.** KEGG pathway analysis of autophagy pathways affected by Mn in mouse ventral midbrain.

**Supplementary Material 6:** Supplementary document

## Data Availability

RNA-seq will be deposited in the Gene Expression Omnibus (GEO) at the time of publication of this study. Quantitative data that support the findings of this study are available in this manuscript and supplementary materials. All other data that support the findings of this study are available from the corresponding authors on reasonable request.
