# Supplementary figures and images for: A partial Drp1 knockout improves autophagy flux independent of mitochondrial function
Source: Mol Neurodegener. 2024 Mar 19;19:26. doi: 10.1186/s13024-024-00708-w (PMC10953112; doi:10.1186/s13024-024-00708-w)

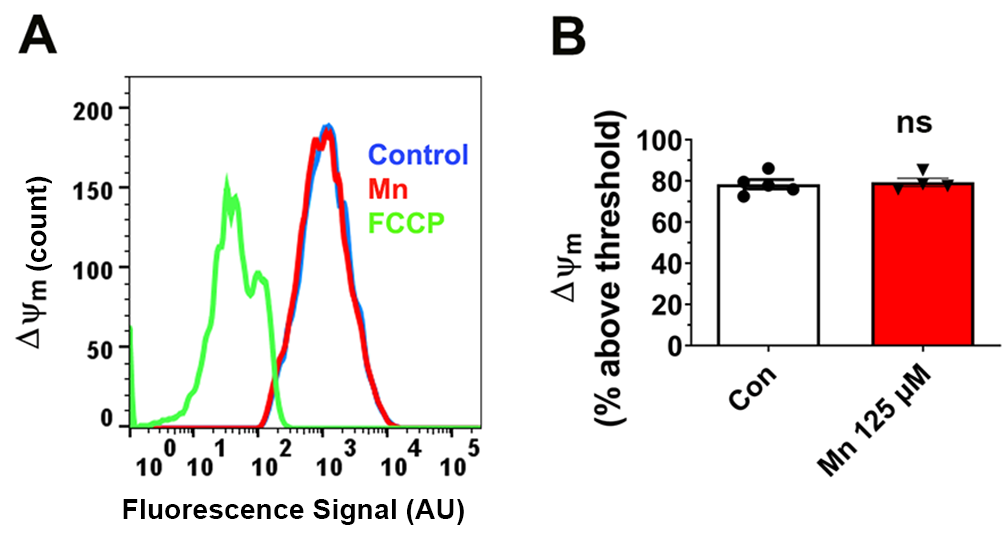

Supplement: Supplementary file 1 — Supplementary Material 1: Fig. S1. Effects of 125 µM Mn treatment on mitochondrial membrane potential [file 13024_2024_708_MOESM2_ESM.tif]

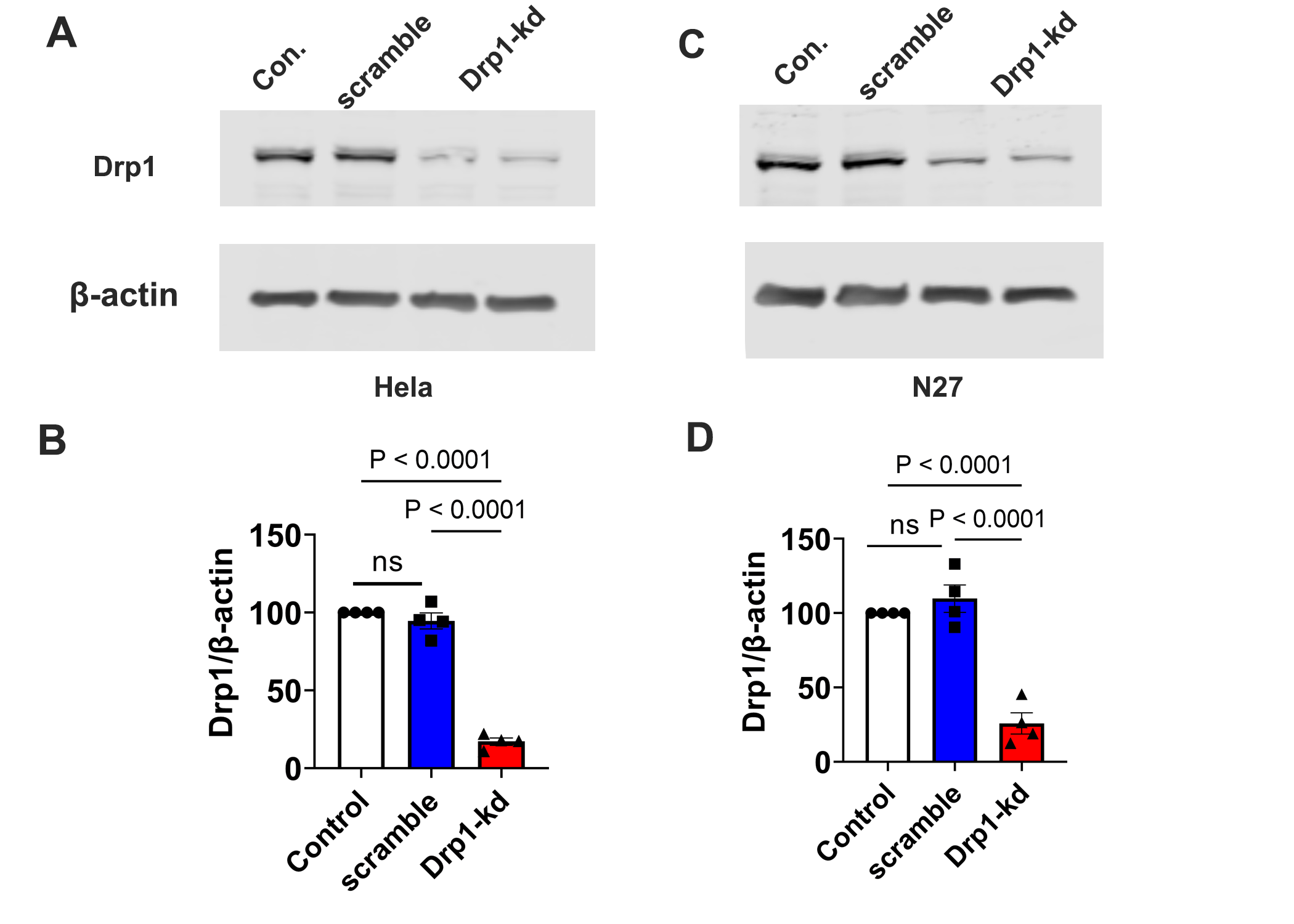

Supplement: Supplementary file 2 — Supplementary Material 2: Fig. S2. Efficiency of siRNA-mediated Drp1-KD in HeLa and N27 cells [file 13024_2024_708_MOESM3_ESM.tif]

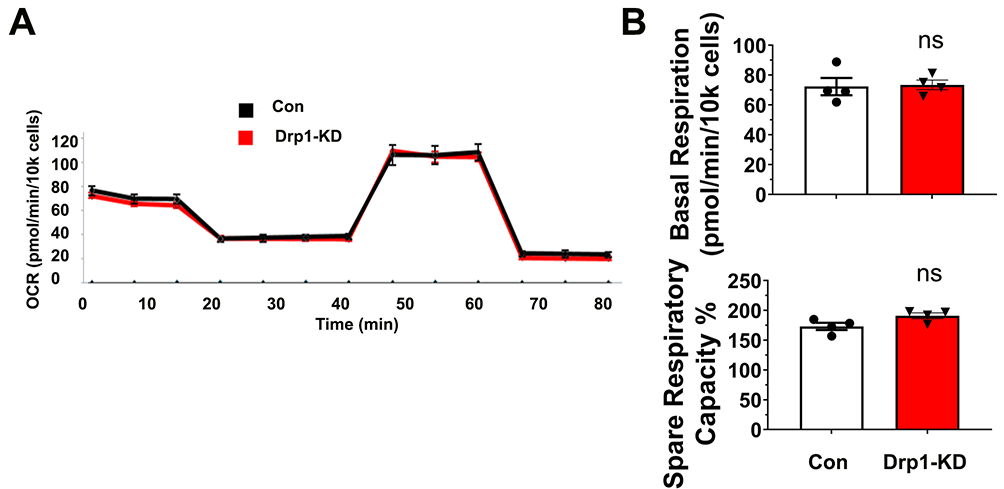

Supplement: Supplementary file 3 — Supplementary Material 3: Fig. S3. Effects of Drp1 knockdown on mitochondrial respiration [file 13024_2024_708_MOESM4_ESM.tif]

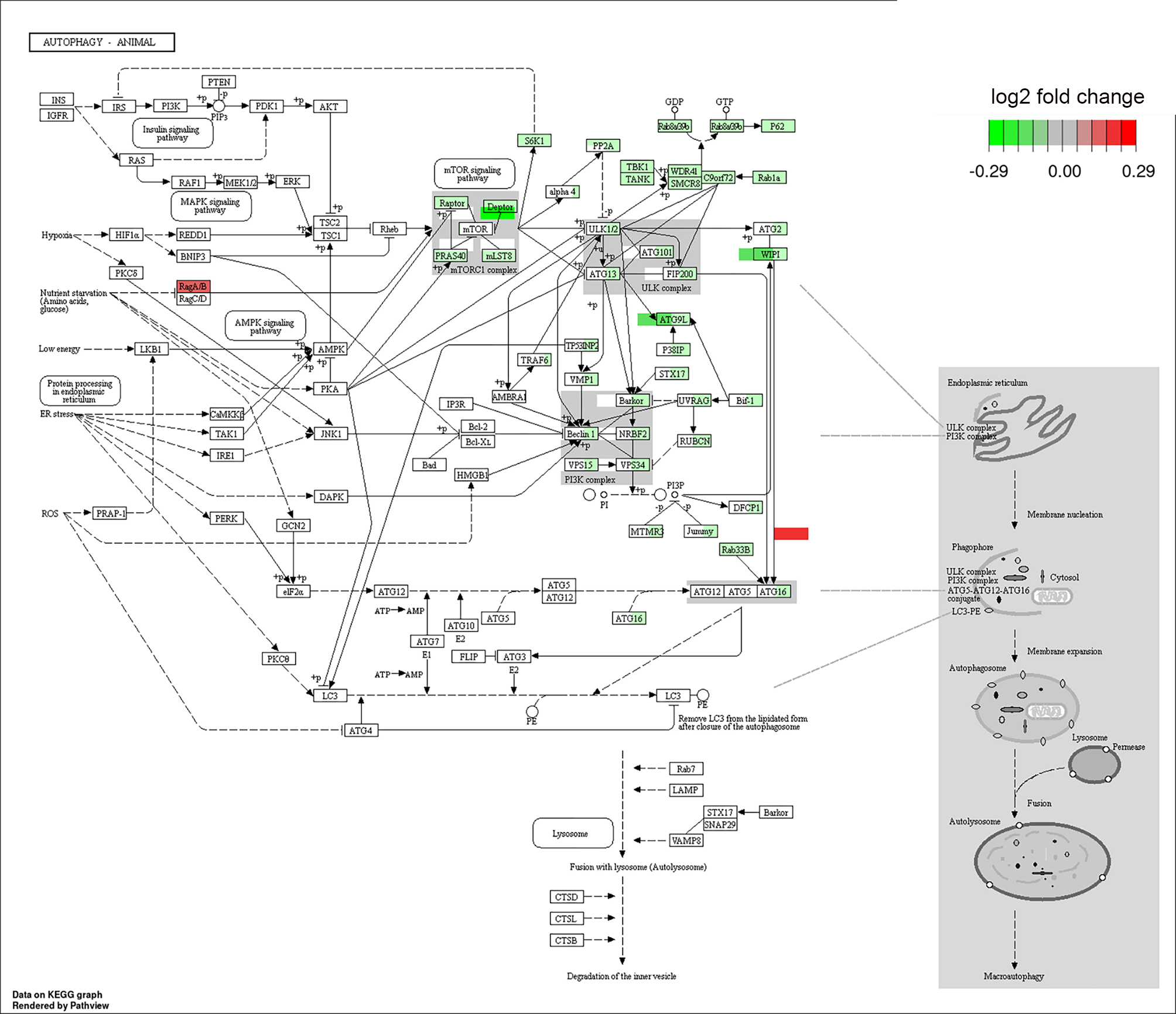

Supplement: Supplementary file 5 — Supplementary Material 5: Fig. S5. KEGG pathway analysis of autophagy pathways affected by Mn in mouse ventral midbrain. [file 13024_2024_708_MOESM5_ESM.tif]
