# Supplementary material for: A partial Drp1 knockout improves autophagy flux independent of mitochondrial function
Source: Mol Neurodegener. 2024 Mar 19;19:26. doi: 10.1186/s13024-024-00708-w (PMC10953112; doi:10.1186/s13024-024-00708-w)
Supplement: Supplementary file 6 — Supplementary Material 6: Supplementary document [file 13024_2024_708_MOESM6_ESM.docx]

**Supplementary Materials**

**A partial Drp1 knockout improves autophagy flux independent of mitochondrial function**

**Rebecca Z. Fan^1,†^, Carolina Sportelli^1,†^, Yanhao Lai^1^, Said S. Salehe^1^, Jennifer R. Pinnell^1^, Harry J. Brown, Jason R. Richardson^1^, Shouqing Luo^2^, Kim Tieu^1,3,*^.**

^1^Department of Environmental Health Sciences, Florida International University, Miami, USA

^2^Peninsula Schools of Medicine and Dentistry, Plymouth University, Plymouth, UK

^3^Biomolecular Sciences Institute Florida International University

† Contributed equally to this study

^*^To whom correspondence may be addressed: Kim Tieu (ktieu@fiu.edu)

**This supplementary file includes:**

Supplementary Material 1: Fig. S1. Effects of 125 µM Mn treatment on mitochondrial membrane potential

Supplementary Material 2: Fig. S2. Efficiency of siRNA-mediated Drp1-KD in HeLa and N27 cells

Supplementary Material 3: Fig. S3. Effects of Drp1 knockdown on mitochondrial respiration

Supplementary Material 4: Supplementary file 1 - RNAseq data

Supplementary Material 5: Fig. S5. KEGG pathway analysis of autophagy pathways affected by Mn in mouse ventral midbrain.


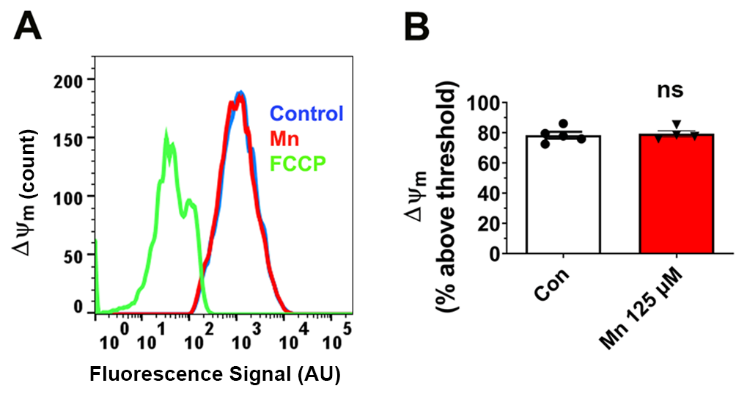


**Fig. S1** Mn treatment does not reduce mitochondrial membrane potential in dopaminergic neuronal cells. (**A**) N27 cells were treated with 125µM Mn for 24h before mitochondrial membrane potential (ΔΨm) was assessed using TMRM (50nM), and fluorescent intensity was analyzed using flow cytometry as previously described [13]. The uncoupler agent carbonyl cyanide 4- (trifluoromethoxy) phenylhydrazone (FCCP, 20μM) was used as positive control to collapse ΔΨm to establish the threshold. (**B**) Signal intensity (AU, arbitrary unit) was expressed as % above this threshold. Data represents mean ± SEM (n=3 independent experiments), student t-test was used. No collapse of membrane potential was observed in the Mn treated group as compared to control (P=0.7552).


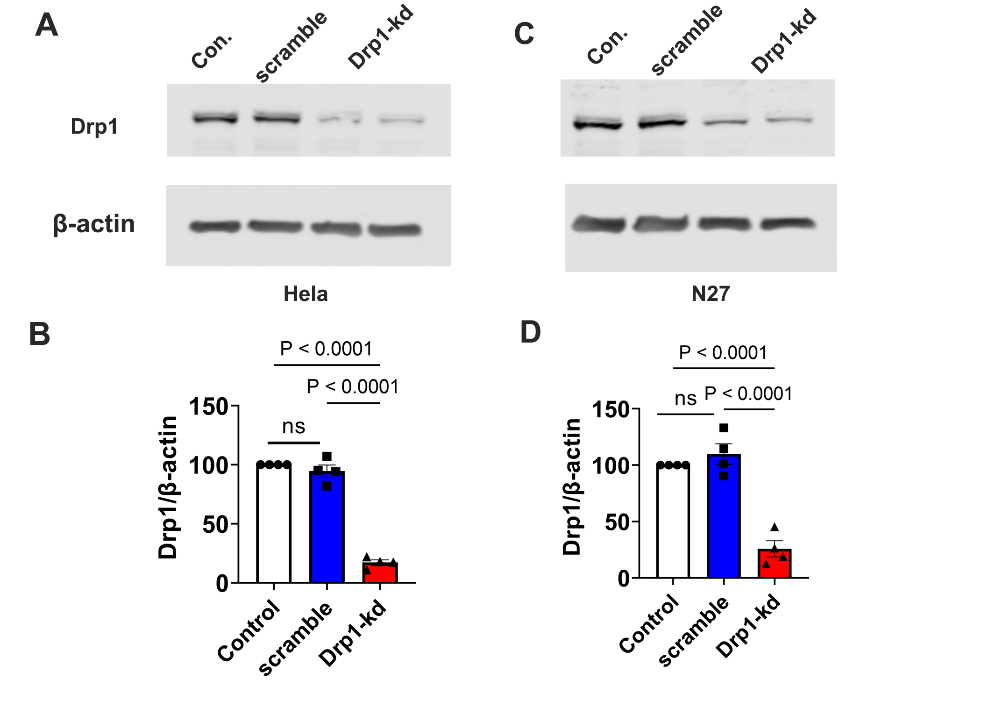


**Fig. S2** Drp1-knockdown (KD) in HeLa and N27 cells. SMARTpool: siGENOME Human *DNM1L* siRNA was used for HeLa cells, and SMARTpool: siGENOME Rat *Dnm1l* siRNA was used for N27 cells. Cells were transfected with these siRNAs for 48h before processed for immunoblotting. Representative western blot images (**A**) and quantification results (**B**) for Hela cell. Representative western blot images (**C**) and quantification results (**D**) for N27 cell. Data represents Mean ± SEM, n=3 independent experiment. Data analyzed by one-way ANOVA, followed by Tukey post hoc test.


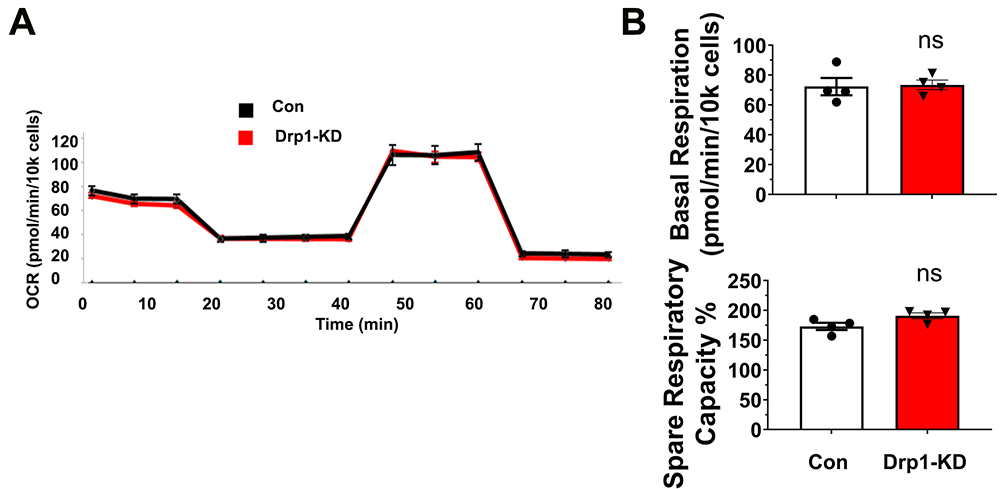


**Fig. S3** A partial Drp1 knockdown did not alter mitochondrial respiration in dopaminergic neuronal cells**.** Mitochondrial respiration was assessed by measuring OCR using the XF^e^96 Extracellular Flux Analyzer. (**A**) Representative kinetic graph showing mitochondrial respiration. (**B**) Drp1 knockdown did not significantly change Basal Respiration (P=0.8720) or Spare Respiratory Capacity% (student t-test, P=0.0616). Data represent mean ± SEM, n=4 independent experiments, 8 replicates per group for each run.

| 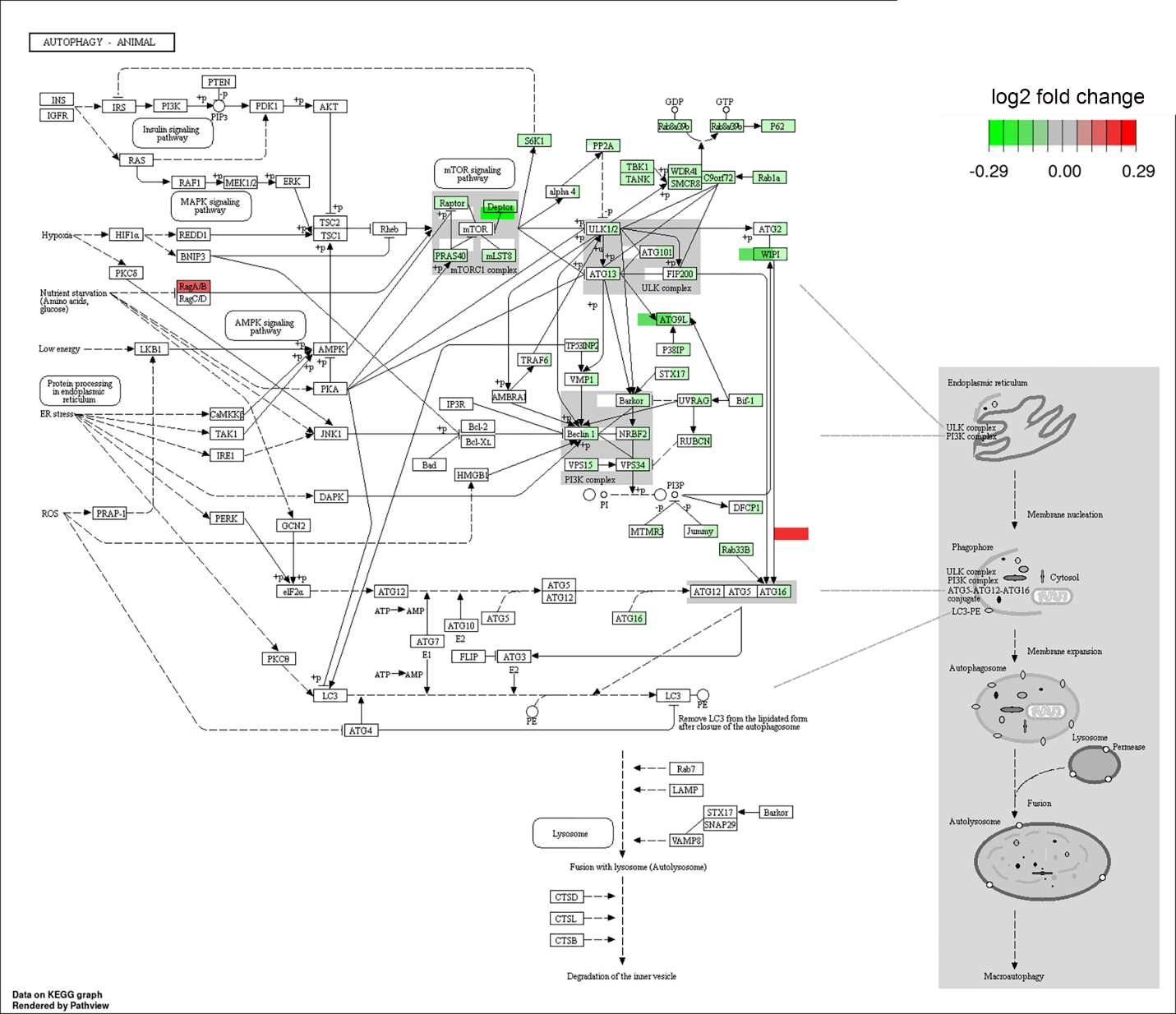 |
| --- |

**Fig. S5** KEGG pathways analysis of the autophagy pathways in the ventral midbrain of Mn treated mice. KEGG-Autophagy animal (mmu04140) pathway analysis was used to examine whether the autophagy pathways would be either up-regulated or down-regulated after exposure to Mn. The differentially expressed genes (DEGs) that were upregulated are shown in red and the DEGs that were downregulated are green. Among these DEGs, Rab33b (p=0.0077, Log2Fold Change=0.1797) and Rraga (p=0.0415, Log2Fold Change=0.1233) were significantly upregulated, whereas Wipi1 (p=0.0235, Log2Fold Change=-0.1469), Deptor (p=0.0313, Log2Fold Change= -0.2915) and Atg9a (p=0.0483, Log2Fold Change= -0.1478) were significantly downregulated.
